# Identification of the Functions and Prognostic Values of RNA Binding Proteins in Bladder Cancer

**DOI:** 10.3389/fgene.2021.574196

**Published:** 2021-06-22

**Authors:** Yue Wu, Zheng Liu, Xian Wei, Huan Feng, Bintao Hu, Bo Liu, Yang Luan, Yajun Ruan, Xiaming Liu, Zhuo Liu, Shaogang Wang, Jihong Liu, Tao Wang

**Affiliations:** ^1^Department of Urology, Tongji Hospital, Tongji Medical College, Huazhong University of Science and Technology, Wuhan, China; ^2^Institute of Urology, Tongji Hospital, Tongji Medical College, Huazhong University of Science and Technology, Wuhan, China; ^3^Department of Oncology, Tongji Hospital, Tongji Medical College, Huazhong University of Science and Technology, Wuhan, China

**Keywords:** bladder cancer, RNA binding proteins, prognostic model, overall survival, bioinformatics

## Abstract

Post-transcriptional regulation plays a leading role in gene regulation and RNA binding proteins (RBPs) are the most important posttranscriptional regulatory protein. RBPs had been found to be abnormally expressed in a variety of tumors and is closely related to its occurrence and progression. However, the exact mechanism of RBPs in bladder cancer (BC) is unknown. We downloaded transcriptomic data of BC from the Cancer Genome Atlas (TCGA) database and used bioinformatics techniques for subsequent analysis. A total of 116 differentially expressed RBPs were selected, among which 61 were up-regulated and 55 were down-regulated. We then identified 12 prognostic RBPs including *CTIF, CTU1, DARS2, ENOX1, IGF2BP2, LIN28A, MTG1, NOVA1, PPARGC1B, RBMS3, TDRD1*, and *ZNF106*, and constructed a prognostic risk score model. Based on this model we found that patients in the high-risk group had poorer overall survival (*P* < 0.001), and the area under the receiver operator characteristic curve for this model was 0.677 for 1 year, 0.697 for 3 years, and 0.709 for 5 years. Next, we drew a nomogram based on the risk score and other clinical variables, which showed better predictive performance. Our findings contribute to a better understanding of the pathogenesis, progression and metastasis of BC. The model of these 12 genes has good predictive value and may have good prospects for improving clinical treatment regimens and patient prognosis.

## Introduction

Bladder cancer (BC) is one of the most common malignant tumors worldwide and the most common malignant tumor in the urinary system, with more than 400,000 new cases diagnosed and 160,000 deaths annually (Torre et al., 2015; Antoni et al., 2017). BC is a heterogeneous tumor with two subtypes: non-muscle-invasive BC (NMIBC) and muscle-invasive BC (MIBC). NMIBC rarely progresses, but half of patients develop tumor recurrence within 5 years, and 20–30% of NMIBC patients progress to MIBC (Chamie et al., 2013). MIBC progresses frequently, and the 5-year survival rate is as low as 8.1% (Abdollah et al., 2013). Although multiple studies have revealed potential biomarkers and therapeutic targets for BC (Cancer Genome Atlas Research Network, 2014; Knowles and Hurst, 2015; Pietzak et al., 2017), and significant advances have been made in surgical techniques and adjuvant chemotherapy, the mortality rate for advanced BC remains high, and the pathogenesis and progression of BC remain unclear (Siegel et al., 2015). Therefore, further understanding of the molecular mechanisms of BC occurrence, progression, and invasion may improve the early detection and diagnosis of BC.

Post-transcriptional regulation plays a leading role in gene regulation, and RNA-binding proteins (RBPs) are the most well-known post-transcriptional regulators. RBPs are widely expressed in cells and can bind to and function with a variety of RNA types, including mRNAs, tRNAs, rRNAs, ncRNAs, snRNAs, miRNAs, and snoRNAs (New et al., 2019; Otsuka et al., 2019). Currently, 1542 human RBP genes have been experimentally verified, accounting for 7.5% of all protein-coding genes (Gerstberger et al., 2014). By binding to RNA, RBPs form ribonucleoprotein complexes to regulate cell metabolism and coordinate the maturation, transportation, stabilization, and degradation of various RNAs (Gerstberger et al., 2014). RBPs have been associated with various human diseases. Defects in ribosomal proteins and rRNA biogenic factors in RBPs severely affect bone marrow and skin functions, leading to Diamond-Blackfan anemia and Shwachman-Diamond syndrome (Narla and Ebert, 2010). The occurrence of various neuromuscular diseases is related to abnormal RNA or protein aggregation due to mutations in RBPs (Cooper et al., 2009). However, studies on the role of RBPs in tumors remain rare.

Several studies have shown that RBPs are abnormally expressed in tumor tissues and are associated with patient prognosis (Patry et al., 2003; King et al., 2011; Wurth et al., 2016). However, few RBPs have been thoroughly studied and identified as having key roles in tumors. One study showed that antagonizing HuR protein expression significantly reduced the proliferation of ovarian, cervical, breast, and colon cancer cells (Abdelmohsen et al., 2008). Additionally, ZEB1 inhibits epithelial splice regulatory protein 1 mRNA expression, leading to increased invasiveness in lung, breast and pancreatic cancer cells (Preca et al., 2015). Therefore, in this study, we systematically analyzed the molecular function and clinical significance of RBPs in BC to fully understand the role of RBPs in BC and aid in developing potential therapeutic targets and biomarkers.

## Materials and Methods

### Preprocessing Data and Identifying Differential RBP Expression

Transcriptomic data from 19 normal bladder tissue samples and 411 BC tissue samples were downloaded from The Cancer Genome Atlas (TCGA) database https://portal.gdc.cancer.gov/). We used the edgeR package (http://www.bioconductor.org/packages/release/bioc/html/edgeR.html) to preprocess raw data, remove genes with average expression values <1 and standardize the data. We identified the differentially expressed RBPs based on |log_2_ FC| >1.0 and a false discovery rate (FDR) <0.05.

### Functional Enrichment Analysis

We performed a comprehensive pathway and functional enrichment analysis using the WEB-based Gene Set Analysis Toolkit (WebGestalt, http://www.webgestalt.org/) online analysis tool. The Gene Ontology (GO) terms, including cellular component, biological process, and molecular function, as well as the Kyoto Encyclopedia of Genes and Genomes (KEGG) pathways, were enriched using the WebGestalt online tool. Only FDR values and *P*-values <0.05 were considered significant.

### Screening for Prognostic-Related RBPs

We identified prognostic-related RBPs using the survival R package for univariate Cox regression analysis of differentially expressed RBPs. We performed the least absolute shrinkage and selection operator (LASSO) algorithm on significant RBPs to further screen for their prognostic significance. Finally, we used multivariate Cox regression analysis to further screen RBPs with prognostic value. *P* < 0.05 was considered significant.

### Construction and Evaluation of the Prognostic Model

We constructed a predictive model based on the RBPs screened via the multivariate Cox regression analysis. With this model, we calculated each patient's risk score according to the following formula:

$$\begin{array}{l} \text{Risk score}=\overset{n}{\underset{i=1}{\sum }}Expi\beta i, \end{array}$$

where β is the regression coefficient of each prognostic gene, and Exp is the expression value of the corresponding gene. We then divided the BC patients from TCGA into high-risk and low-risk groups based on their median risk score and compared the differences in overall survival (OS) between both groups to evaluate the predictive efficacy of the model. We used the survivalROC R package to draw receiver operating characteristic (ROC) curves to assess the model's predictive performance. To further verify the model's predictive power, we downloaded a GSE13507 dataset containing information on 256 samples from the Gene Expression Omnibus (GEO, https://www.ncbi.nlm.nih.gov/geo/) database as a validation cohort.

### Correlation Between the Prognostic-Related Model and Clinical Variables

To explore the clinical significance of the prognostic-related model for different clinical variables, we stratified patients according to clinical parameters and performed survival analysis. We also explored the relationship between eight prognostic RBPs and clinical variables. *P* < 0.05 was considered significant.

### Independence of the RBP-based Prognostic Model From Patients' Clinical Characteristics

We performed univariate and multivariate Cox regression analyses based on clinical characteristics including age, sex, tumor grade, tumor stage, T stage, N stage, and M stage in patients with BC to determine whether the RBP-based prognostic model was an independent prognostic factor. We used the rms R package to generate a nomogram to predict the OS, and we performed Kaplan-Meier survival and ROC curve analyses of the nomogram based on TCGA and GEO patient data to evaluate its clinical applicability. *P* < 0.05 was considered significant.

### Exploration of the Regulatory Network of Prognostic-Related RBPs and Its Relationship With Immune Cell Infiltration

We downloaded the transcription factors associated with tumorigenesis and progression from the CISTROME project (www.cistrome.org) to investigate the role of the prognostic gene regulatory network. We then performed coexpression analysis to explore the expression regulation relationship between transcription factors and prognostic genes. Additionally, we calculated the abundance of infiltrating immune cells in each sample using a deconvolution algorithm and based on data from 22 groups of genes related to infiltrating immune cells. We used CIBERSORT and its supplied gene set, LM22, to estimate the degree of immune cell infiltration in different clusters. *P* < 0.05 was considered significant.

### Expression Level and Prognostic Significance Verification of Prognostic-Related RBPs

We used the Kaplan-Meier plotter online tool (https://kmplot.com/analysis/) to assess the prognostic significance of these prognostic-related RBPs in patients with BC. We used the Human Protein Atlas (HPA, https://www.proteinatlas.org/) online database to verify the protein expression levels of these prognostic-related RBPs.

### Cell Culture

SVHUC1, J82, T24, 5637, and RT4 cells were purchased from the Type Culture Collection of the Chinese Academy of Sciences (Shanghai, China). SVHUC1 is a normal bladder epithelial cell that serves as an experimental control. RT4 is a low-grade non-invasive bladder cancer cell, 5637 is a moderately invasive bladder cancer cell, while T24 and J82 are both high-grade and highly invasive bladder cancer cells. SVHUC1 cells were cultured in F12K medium (N3520) (Sigma-Aldrich, St. Louis, Missouri, USA) containing 10% fetal bovine serum (FBS) (Gibco, Grand Island, NY, USA); J82, T24, and 5637 cells were cultured in RPMI-1640 containing 10% FBS; RT4 cells were cultured in McCOY's 5A (M4892) (Sigma-Aldrich, St. Louis, Missouri, USA) containing 10% FBS in a humidified atmosphere of 5% CO_2_ at 37°C. The medium is renewed every 2–3 days. The experiments were then carried out on passage 3–5 cells.

### Real-Time Quantitative Polymerase Chain Reaction Verification

Total RNA was extracted with Trizol reagent (Beyotime, Jiangsu, China). The total RNA was reverse transcribed into cDNA using the PrimeScript™ RT reagent Kit (Perfect Real Time) (TaKaRa, Japan) and RT-QPCR assay was performed using the TB Green® Premix Ex Taq™ II (Tli RNaseH Plus) (TaKaRa, Japan) in an ABI Prism 7300 system (Thermo Fisher Scientific). The GAPDH was used as the reference gene and 2^−Δ*ΔCt*^ method was used to calculate the fold change of the target gene. All primers sequences used in the study were shown in Supplementary Table 1.

## Results

### Identifying Differentially Expressed RBPs in BC Patients

**Figure 1:**
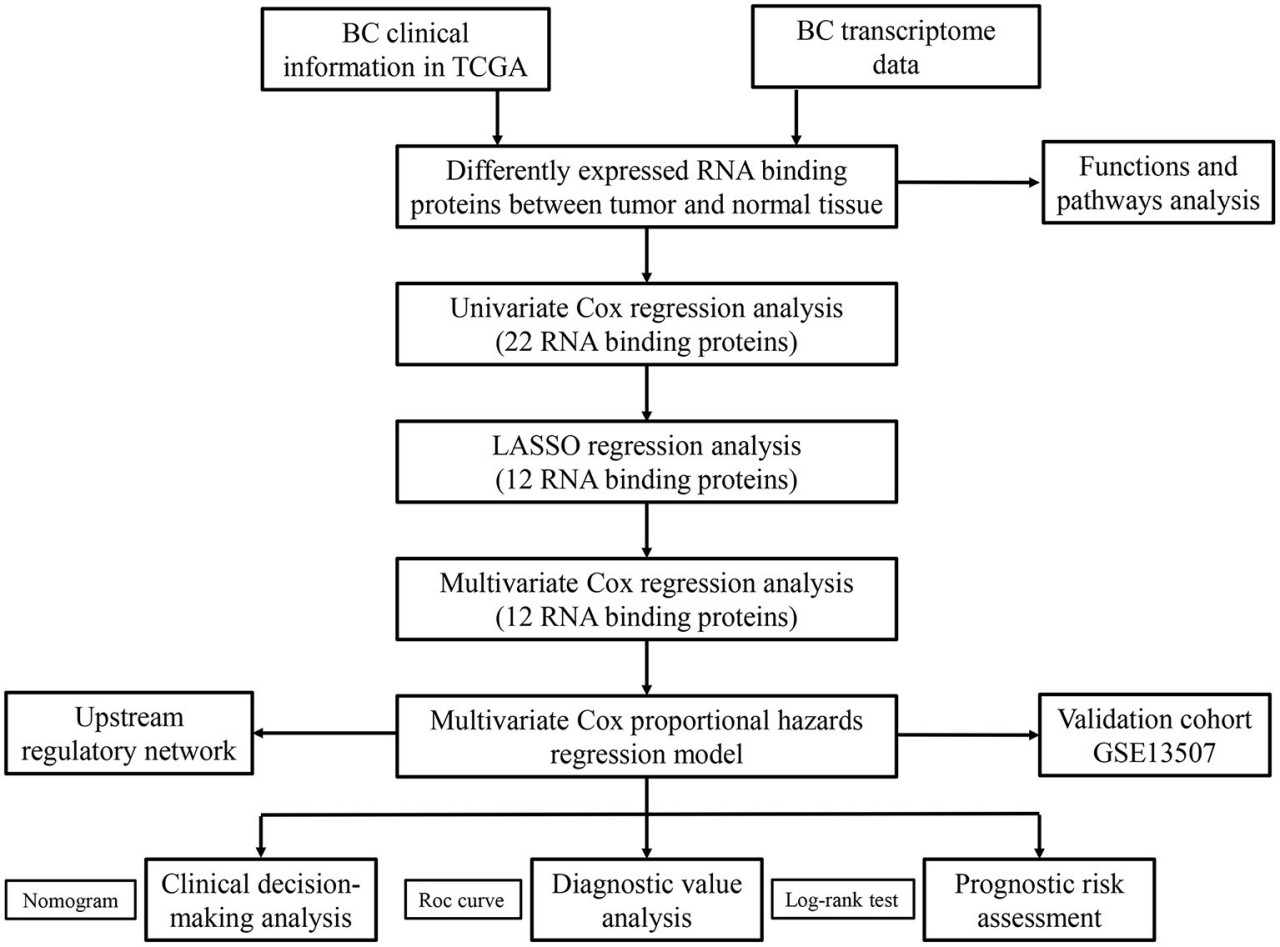
The flow chart for analyzing the RBPs in BC.

**Figure 2:**
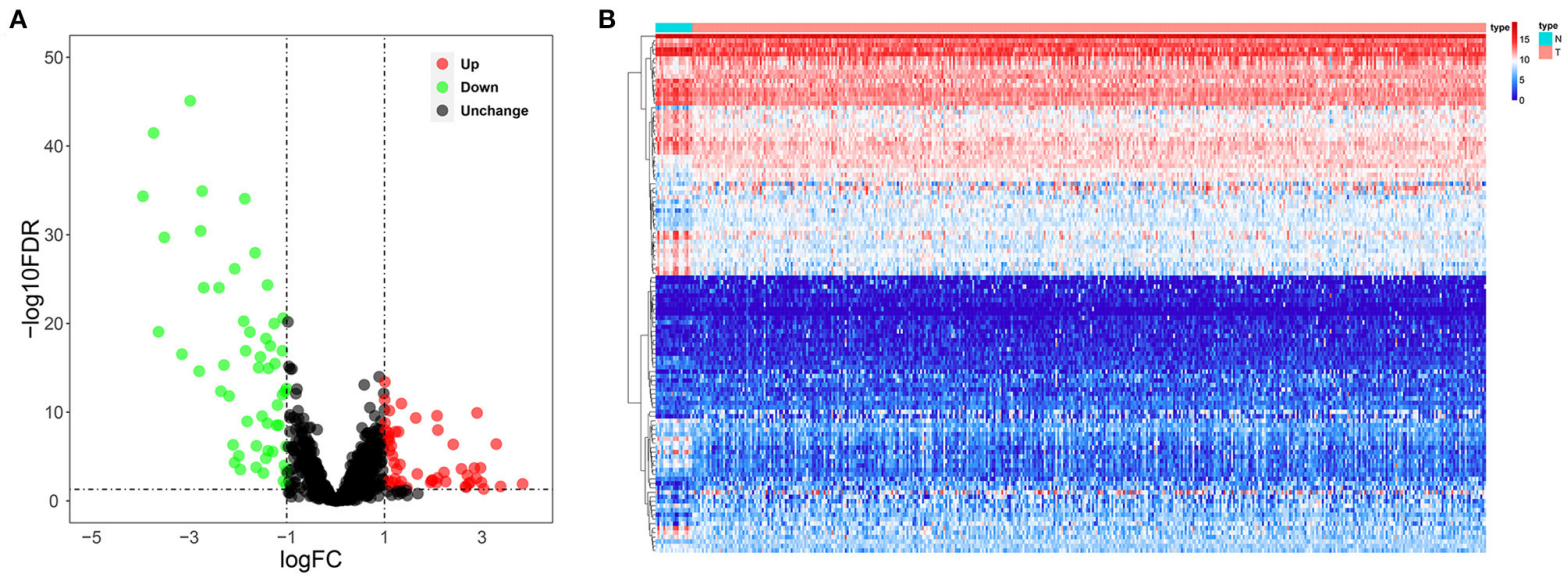
The differentially expressed RBPs in BC. **(A)** Volcano plot; **(B)** Heat map.

We systematically analyzed the roles of RBPs in patients with BC. Figure 1 shows the research flow chart. The BC patients' transcriptomic data were downloaded from TCGA, including 19 normal bladder tissue samples and 411 tumor tissue samples. We used edgeR software to process the data and identify the differentially expressed RBPs. As per the study criteria (|log_2_ FC| >1.0, FDR < 0.05), we selected 116 differentially expressed RBPs from 1,542 RBPs (Gerstberger et al., 2014). Of these, 61 were upregulated, and 55 were downregulated. Figure 2 shows the expressions and distributions of these differentially expressed RBPs.

### GO and KEGG Enrichment Analysis of Differentially Expressed RBPs

To investigate the biological functions and molecular mechanisms of these differentially expressed RBPs, we conducted GO and KEGG functional enrichment analysis of upregulated and downregulated RBPs through the WebGestalt online tool (Table 1). The biological processes indicated that the upregulated RBPs were significantly enriched in gene silencing, posttranscriptional regulation of gene expression, regulation of gene expression, epigenetics, DNA modification, RNA catabolic processes, regulation of cellular amide metabolic processes, cellular processes involved in reproduction in multicellular organisms, methylation, dsRNA processing, and RNA modification. The downregulated RBPs were significantly enriched in mRNA processing, regulation of mRNA metabolic processes, posttranscriptional regulation of gene expression, regulation of cellular amide metabolic processes, RNA splicing, cytoplasmic translation, RNA catabolic processes, response to oxygen levels, epithelial cell apoptotic processes, and response to ischemia. The upregulated and downregulated RBPs were both enriched in the cellular component associated with the ribonucleoprotein granule. Regarding molecular functions, the upregulated RBPs were significantly enriched in catalytic activity, acting on RNA, mRNA binding, translation regulator activity, nucleotidyltransferase activity, pre-mRNA binding, double-stranded RNA binding, nuclease activity, ribonucleoprotein-complex binding, lipopolysaccharide binding, and regulatory RNA binding. The downregulated RBPs were significantly enriched in mRNA binding, AU-rich-element binding, translation factor activity, RNA binding, translation regulator activity, single-stranded RNA binding, snRNA binding, and ribonucleoprotein-complex binding. KEGG analysis indicated that the upregulated RBPs were significantly enriched in microRNAs in cancer, and the downregulated RBPs were significantly enriched in progesterone-mediated oocyte maturation and oocyte meiosis.

**Table 1:** KEGG pathway and GO enrichment analysis of differentially expressed RBPs.

	**GO term**	***P-*value**	**FDR**
Up-regulated RBPs	Gene silencing	1.53E-13	1.30E-10
	Posttranscriptional regulation of gene expression	4.95E-12	2.11E-9
	Regulation of gene expression, epigenetic	6.75E-11	1.91E-8
	DNA modification	1.08E-10	2.30E-8
	RNA catabolic process	1.66E-9	2.82E-7
	Regulation of cellular amide metabolic process	6.50E-9	9.21E-7
	Cellular process involved in reproduction in Multicellular organism	2.41E-8	2.64E-7
	Methylation	2.48E-8	2.64E-7
	dsRNA processing	6.70E-7	6.33E-5
	RNA modification	9.50E-7	8.08E-5
	Ribonucleoprotein granule	9.13E-10	1.57E-7
	Catalytic activity, acting on RNA	9.88E-15	2.79E-12
	mRNA binding	1.35E-9	1.91E-7
	Translation regulator activity	1.11E-7	1.04E-5
	Nucleotidyltransferase activity	6.98E-7	4.92E-5
	Pre-mRNA binding	8.30E-6	4.68E-5
	Double-stranded RNA binding	1.21E-5	5.67E-4
	Nuclease activity	1.51E-5	6.09E-4
	Ribonucleoprotein complex binding	1.53E-4	0.005
	Lipopolysaccharide binding	3.00E-4	0.009
	Regulatory RNA binding	4.18E-4	0.012
	MicroRNAs in cancer	5.45E-5	0.018
Down-regulated RBPs	mRNA processing	<0.001	<0.001
	Regulation of mRNA metabolic process	1.36E-13	4.64E-11
	Posttranscriptional regulation of gene expression	1.64E-13	4.64E-11
	Regulation of cellular amide metabolic process	1.26E-12	2.67E-10
	RNA splicing	5.92E-11	1.01E-8
	Cytoplasmic translation	6.22E-7	8.81E-5
	RNA catabolic process	1.79E-6	2.17E-4
	Response to oxygen levels	1.28E-4	0.014
	Epithelial cell apoptotic process	3.42E-4	0.030
	Response to ischemia	3.53E-4	0.030
	Ribonucleoprotein granule	5.14E-9	8.84E-7
	mRNA binding	<0.001	<0.001
	AU-rich element binding	5.03E-12	7.09E-10
	Translation factor activity, RNA binding	6.41E-10	6.03E-8
	Translation regulator activity	5.69E-8	4.01E-6
	Single-stranded RNA binding	6.34E-7	3.58E-5
	snRNA binding	3.55E-4	0.017
	Ribonucleoprotein complex binding	0.001	0.045
	Progesterone-mediated oocyte maturation	5.19E-5	0.017
	Oocyte meiosis	1.25E-4	0.020

### Screening for Prognosis-Associated RBPs

**Figure 3:**
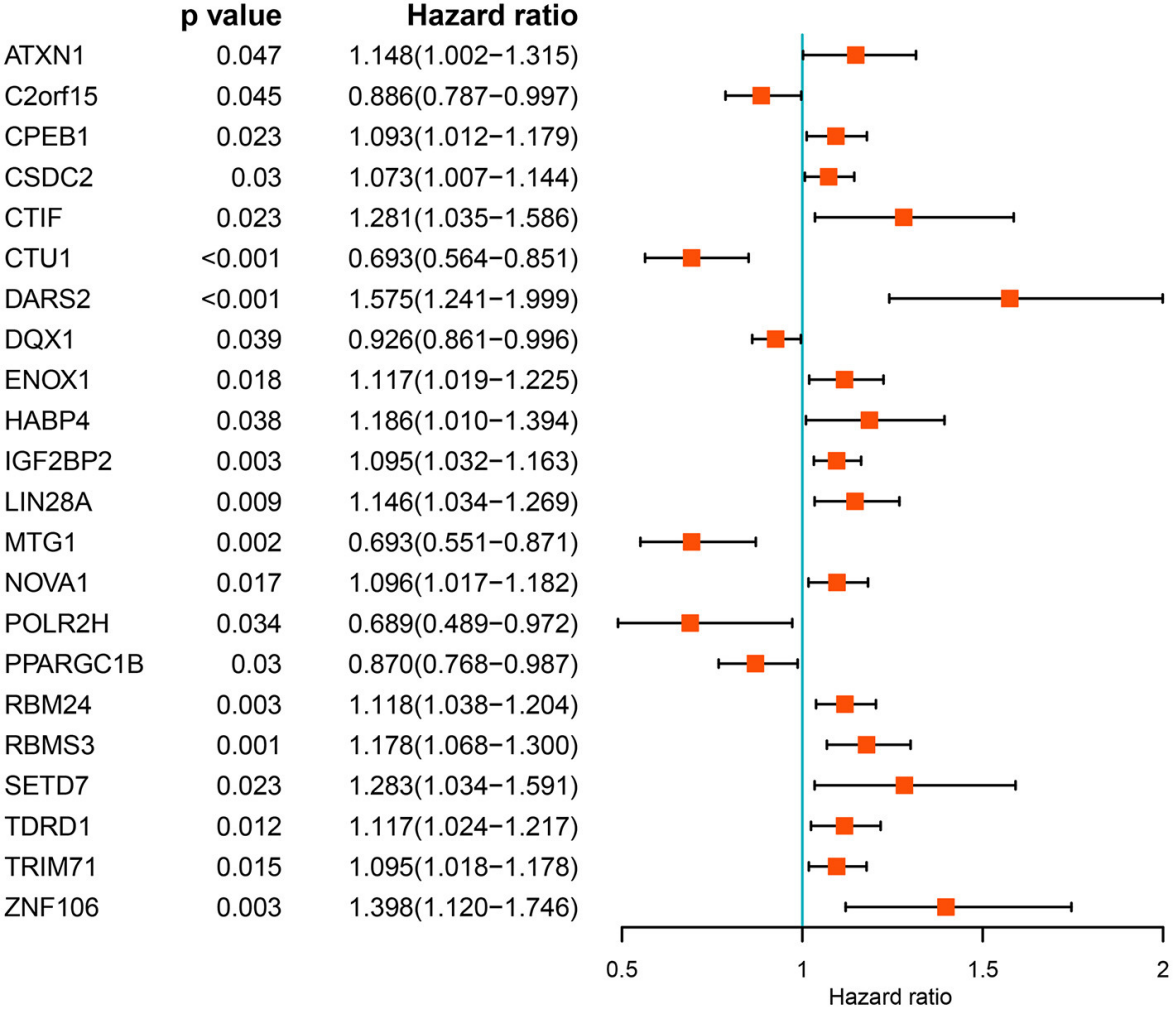
Univariate Cox regression analysis of differentially expressed RBPs.

We performed a univariate Cox regression analysis of all the differentially expressed RBPs to determine their prognostic significance and obtained 22 prognostic RBPs (Figure 3). We then performed LASSO regression analysis on these RBPs and obtained 12 RBPs with prognostic value including *ZNF106, CTIF, RBMS3, NOVA1, PPARGC1B, MTG1, DARS2, CTU1, ENOX1, LIN28A, IGF2BP2*, and *TDRD1* (Supplementary Figure 1). We further performed multivariate Cox regression analysis on the 12 RBPs and finally identified 12 genes for subsequent analysis.

### Constructing and Validating Prognostic-Related Genetic Risk Score Model

We built a predictive model based on the 12 RBPs screened via multivariate Cox regression analysis (Table 2). The risk score for each patient with BC was calculated using the formula, risk score = (0.0678 ^*^ ExpZNF106) + (0.1601 ^*^ ExpCTIF) + (0.0642 ^*^ ExpRBMS3) + (0.0172 ^*^ ExpNOVA1) + (−0.1390 ^*^ ExpPPARGC1B) + (−0.1443 ^*^ ExpMTG1) + (0.4480 ^*^ ExpDARS2) + (−0.2467 ^*^ ExpCTU1) + (0.0392 ^*^ ExpENOX1) + (0.1514 ^*^ ExpLIN28A) + (0.0384 ^*^ ExpIGF2BP2) + (0.0235 ^*^ ExpTDRD1).

**Table 2:** Multivariate Cox regression analysis to identify prognosis-related hub RNA binding proteins.

**Gene**	**Coef**	**Exp(coef)**	**se(coef)**	**z**	***Pr*(>|z|)**
ZNF106	0.0678	1.0702	0.1555	0.4363	0.6626
CTIF	0.1601	1.1737	0.1299	1.2329	0.2176
RBMS3	0.0642	1.0663	0.0711	0.9037	0.3661
NOVA1	0.0172	1.0173	0.0499	0.3441	0.7308
PPARGC1B	−0.1390	0.8703	0.0765	−1.8161	0.0694
MTG1	−0.1443	0.8656	0.1317	−1.0964	0.2729
DARS2	0.4480	1.5653	0.1340	3.3444	0.0008
CTU1	−0.2467	0.7814	0.1201	−2.0535	0.0400
ENOX1	0.0392	1.0400	0.0601	0.6525	0.5141
LIN28A	0.1514	1.1634	0.0592	2.5571	0.0106
IGF2BP2	0.0384	1.0392	0.0398	0.9653	0.3344
TDRD1	0.0235	1.0238	0.0519	0.4536	0.6501

**Figure 4:**
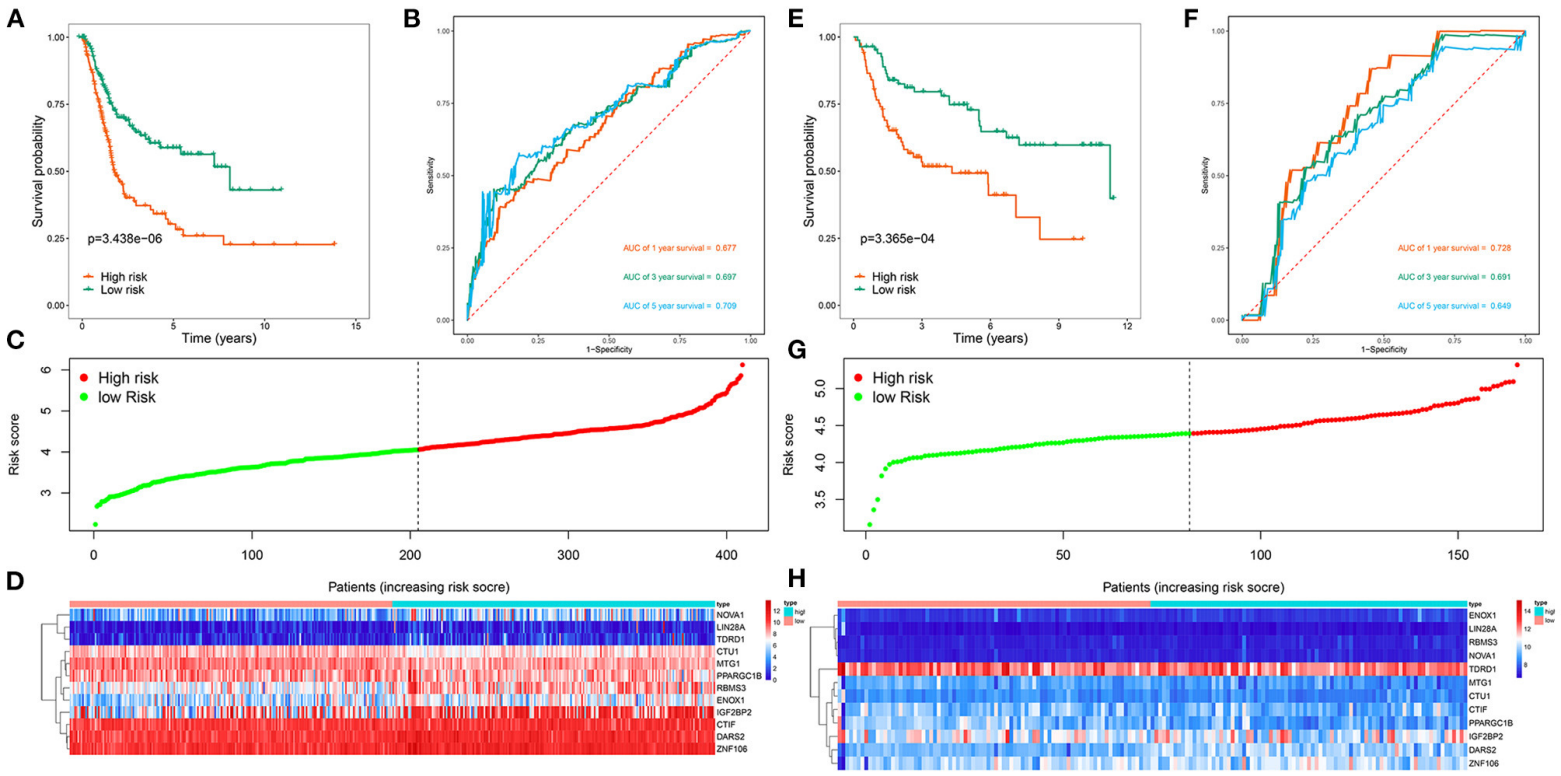
Risk score analysis of 12 genes prognostic model in the TCGA and GSE13507 cohorts. **(A)** Survival curve for high-risk and low-risk groups in the TCGA cohort; **(B)** ROC curves in the TCGA cohort; **(C)** Risk score distribution in the TCGA cohort; **(D)** Expression heatmap in the TCGA cohort; **(E)** Survival curve for high-risk and low-risk groups in the GSE13507 cohort; **(F)** ROC curves in the GSE13507 cohort; **(G)** Risk score distribution in the GSE13507 cohort; **(H)** Expression heatmap in the GSE13507 cohort.

We then divided 411 BC patients into high-risk and low-risk groups based on the median risk score and performed a survival analysis to evaluate their predictive performance. Patients in the high-risk group had poorer OS than did those in the low-risk group (*P* < 0.001; Figure 4A). We further conducted a time-dependent ROC analysis to assess the predictability of this model, and the areas under the ROC curve (AUCs) of this model were 0.677 for 1 year, 0.697 for 3 years, and 0.709 for 5 years (Figure 4B). Figures 4C,D show the risk curve and expression heat map, respectively, of the 12 genes between both groups. We used the same calculation formula for the GSE13507 dataset to evaluate whether the model had similar predictive performances for the other BC patient cohorts. Patients in the high-risk group had a poorer OS (*P* < 0.001, Figure 4E), and the AUC of this model was 0.728 for 1 year, 0.691 for 3 years, and 0.649 for 5 years (Figure 4F). Figures 4G,H show the risk curve and expression heat map, respectively, of the 12 genes between the two groups. The results showed that the model had good predictive performance.

### Prognostic Significance of the Prognostic-Related Model Stratified by Clinical Variables

**Figure 5:**
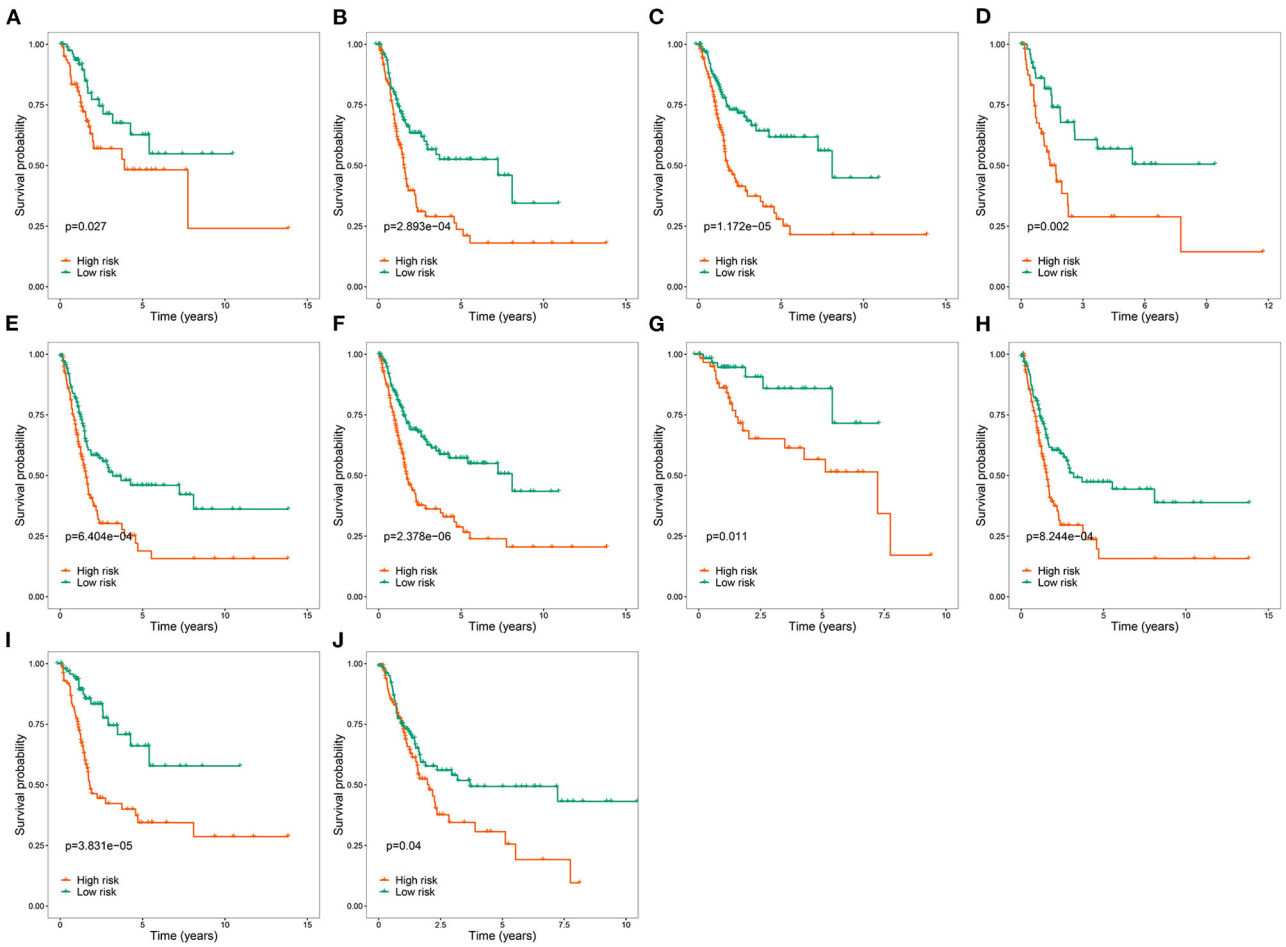
Kaplan-Meier survival curves analysis stratified by different clinical variables. **(A)** Age ≤ 65; **(B)** Age > 65; **(C)** Male; **(D)** Female; **(E)** Stage III–IV; **(F)** High grade; **(G)** T stage 1–2; **(H)** T stage 3–4; **(I)** M0; **(J)** M1-X.

To explore the clinical significance of the RBP-based prognostic model in BC patients stratified by clinical variables, we stratified the patients from TCGA according to age, sex, grade, stage, T stage, N stage, and M stage. Kaplan-Meier survival curve analysis showed that the OS was significantly shorter for patients in the high-risk group than for those in the low-risk group (Figure 5). Therefore, the RBP-based prognostic model can be used to predict the prognosis of BC patients without considering clinical variables.

### Correlation Between the Prognostic-Related Model and Clinical Variables

**Figure 6:**
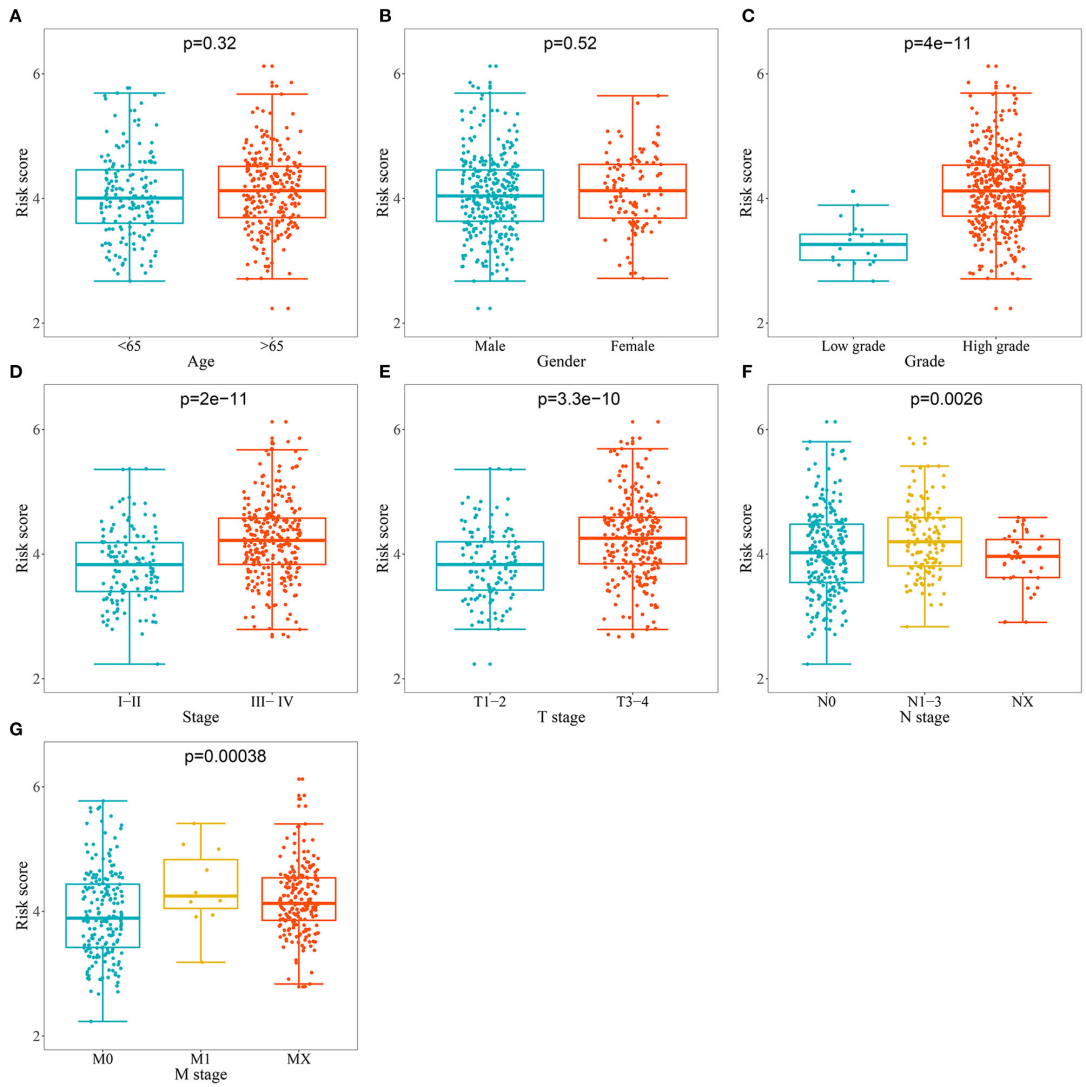
Relationship between prognostic related model and clinical variables. **(A)** Age; **(B)** Gender; **(C)** Grade; **(D)** Stage; **(E)** T stage; **(F)** N stage **(G)** M stage.

We analyzed the correlation between the RBP-based prognostic model and clinical variables to explore whether the prognostic model affected BC tumor progression. We found no significant correlation between age and sex (Figures 6A,B). However, the risk score for low tumor grades was significantly lower than that for high tumor grades (Figure 6C); the risk score for stages I–II was significantly lower than that for stages III–IV (Figure 6D); the risk score for T1–2 was significantly lower than that for T3–4 (Figure 6E); the risk score for N0 was significantly lower than that for N1–3 (Figure 6F), and the risk score for M0 was significantly lower than that for M1 (Figure 6G). These results showed that the prognostic model was significantly correlated with BC tumor progression.

### Correlation Between Prognostic-Related RBPs and Clinical Variables

Based on those results, we analyzed the relationship between prognostic-related RBPs and clinical variables to further investigate the role of prognostic RBPs in BC. DARS2, ENOX1, IGF2BP2, MTG1, PPARGC1B, RBMS3, and ZNF106 were significantly correlated with grade; CTIF, DARS2, ENOX1, IGF2BP2, MTG1, NOVA1, PPARGC1B, RBMS3, and TDRD1 were significantly correlated with stage; DARS2, ENOX1, IGF2BP2, NOVA1, PPARGC1B, RBMS3, TDRD1, and ZNF106 were significantly correlated with T stage; CTIF, IGF2BP2, LIN28A, MTG1, PPARGC1B, and RBMS3 were significantly correlated with M stage; and DARS2 and PPARGC1B were significantly correlated with N stage (Table 3).

**Table 3:** The relationship between prognostic related RBPs and clinicopathologic parameters.

**Gene**		**Grade**		**Stage**		**T stage**		**M stage**		**N stage**	
		**Low**	**High**	**I–II**	**III–IV**	**T1–T2**	**T3–T4**	**M0**	**M1–X**	**N0**	**N1–3**
N		21	386	133	275	124	253	200	208	237	130
CTIF	*t*-value	1.167		2.294		1.803		2.273		1.170	
	*P*-value	0.244		0.022		0.072		0.024		0.243	
CTU1	*t*-value	0.744		0.928		1.311		1.274		1.304	
	*P*-value	0.458		0.354		0.191		0.203		0.193	
DARS2	*t*-value	4.476		3.455		2.837		0.776		2.772	
	*P*-value	<0.001		<0.001		0.005		0.438		0.006	
ENOX1	*t*-value	3.863		2.901		3.931		1.772		1.010	
	*P*-value	<0.001		0.004		<0.001		0.077		0.313	
IGF2BP2	*t*-value	5.098		4.080		4.483		3.903		1.004	
	*P*-value	<0.001		<0.001		<0.001		<0.001		0.316	
LIN28A	*t*-value	1.822		1.553		1.602		2.039		0.074	
	*P*-value	0.069		0.121		0.110		0.042		0.941	
MTG1	*t*-value	2.463		2.481		1.780		2.454		1.886	
	*P*-value	0.014		0.014		0.076		0.015		0.060	
NOVA1	*t*-value	1.399		2.888		3.064		0.103		1.349	
	*P*-value	0.163		0.004		0.002		0.918		0.178	
PPARGC1B	*t*-value	3.601		4.229		3.210		3.450		2.869	
	*P*-value	<0.001		<0.001		0.001		<0.001		0.004	
RBMS3	*t*-value	2.577		5.039		4.562		3.162		1.782	
	*P*-value	0.01		<0.001		<0.001		0.002		0.076	
TDRD1	*t*-value	1.196		2.326		2.929		1.686		1.296	
	*P*-value	0.232		0.021		0.004		0.093		0.196	
ZNF106	*t*-value	2.754		1.002		2.374		0.141		0.763	
	*P*-value	0.006		0.317		0.018		0.888		0.446	

### Establishment and Validation of a Nomogram Based on Clinical Variables

**Figure 7:**
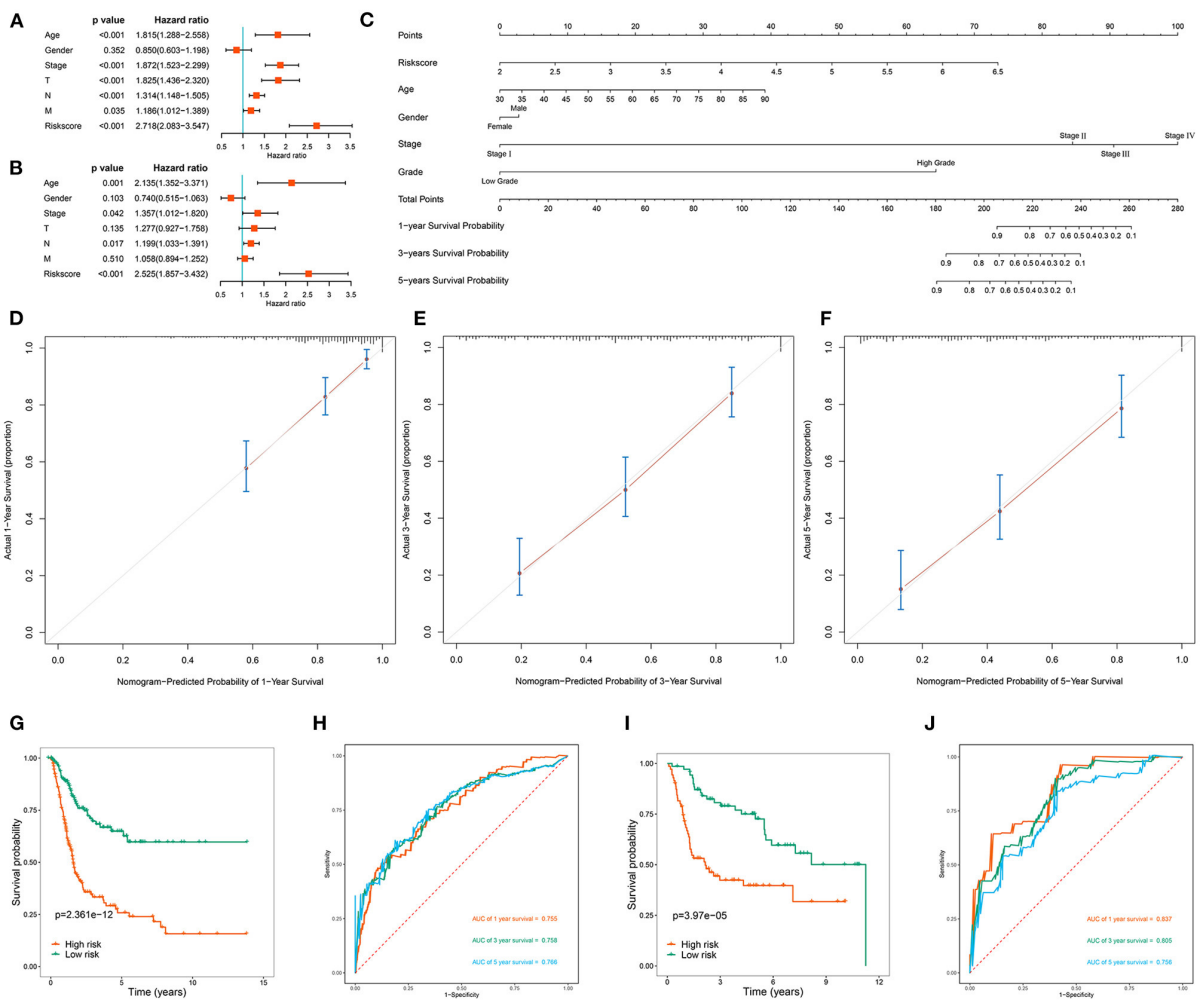
Assessment the prognostic significance of different clinical characteristics and construction of a nomogram in BC patients. **(A)** Univariate Cox regression analysis of correlations between risk score and clinical variables; **(B)** Multivariate Cox regression analysis of correlations between risk score and clinical variables; **(C)** Nomogram for predicting the 1-year, 2-year, and 5-year OS of BC patients; **(D–F)** Calibration curves of the nomogram to predict OS at 1, 3, and 5 years; **(G)** Kaplan-Meier survival analysis of BC patients in TCGA cohort based on the constructed nomogram; **(H)** ROC curves of BC patients in TCGA cohort based on the constructed nomogram; **(I)** Kaplan-Meier survival analysis of BC patients in GSE13507 cohort based on the constructed nomogram; **(J)** ROC curves of BC patients in GSE13507 cohort based on the constructed nomogram.

To clarify whether the RBP-based prognostic model is an independent prognostic factor when other conventional clinical variables are considered in TCGA, we performed univariate regression analysis to assess the predictive values of clinical features in BC patients. Age, tumor stage, primary tumor location, lymph node infiltration, distant metastasis and risk score of BC patients were significantly related to OS (Figure 7A). Subsequently, multivariate regression analysis showed that age (*P* = 0.001), tumor stage (*P* = 0.042), lymph node infiltration (*P* = 0.017), and risk score (*P* < 0.001) were independent prognostic factors associated with OS (Figure 7B). Next, we constructed a nomogram based on the RBP-based prognostic model and other conventional clinical variables to establish a quantitative prognostic evaluation method for BC (Figure 7C). Drawing a vertical line between the prognosis axis and the total point axis enabled estimation of the 1-year, 3-year, and 5-year survival rates for patients with BC. The established calibration curve showed good consistency between the predicted results and the actual results (Figures 7D–F). Kaplan-Meier survival analysis in TCGA showed that the individual risk stratification of patients with BC based on the nomogram better distinguished patients with low survival rates (*P* < 0.001; Figure 7G). The AUCs of the nomogram were 0.755 for 1 year, 0.758 for 3 years, and 0.766 for 5 years (Figure 7H). We also used the GSE13507 dataset to validate the clinical applicability of the nomogram. The nomogram-based risk stratification of patients with BC also better distinguished patients with low survival rates (*P* < 0.001; Figure 7I). The AUCs of the nomogram were 0.837 for 1 year, 0.805 for 3 years, and 0.758 for 5 years (Figure 7J). These results showed that the nomogram had high predictive ability and accuracy for the survival status of patients with BC.

### Upstream Regulatory Network of Prognostic-Related RBPs

**Figure 8:**
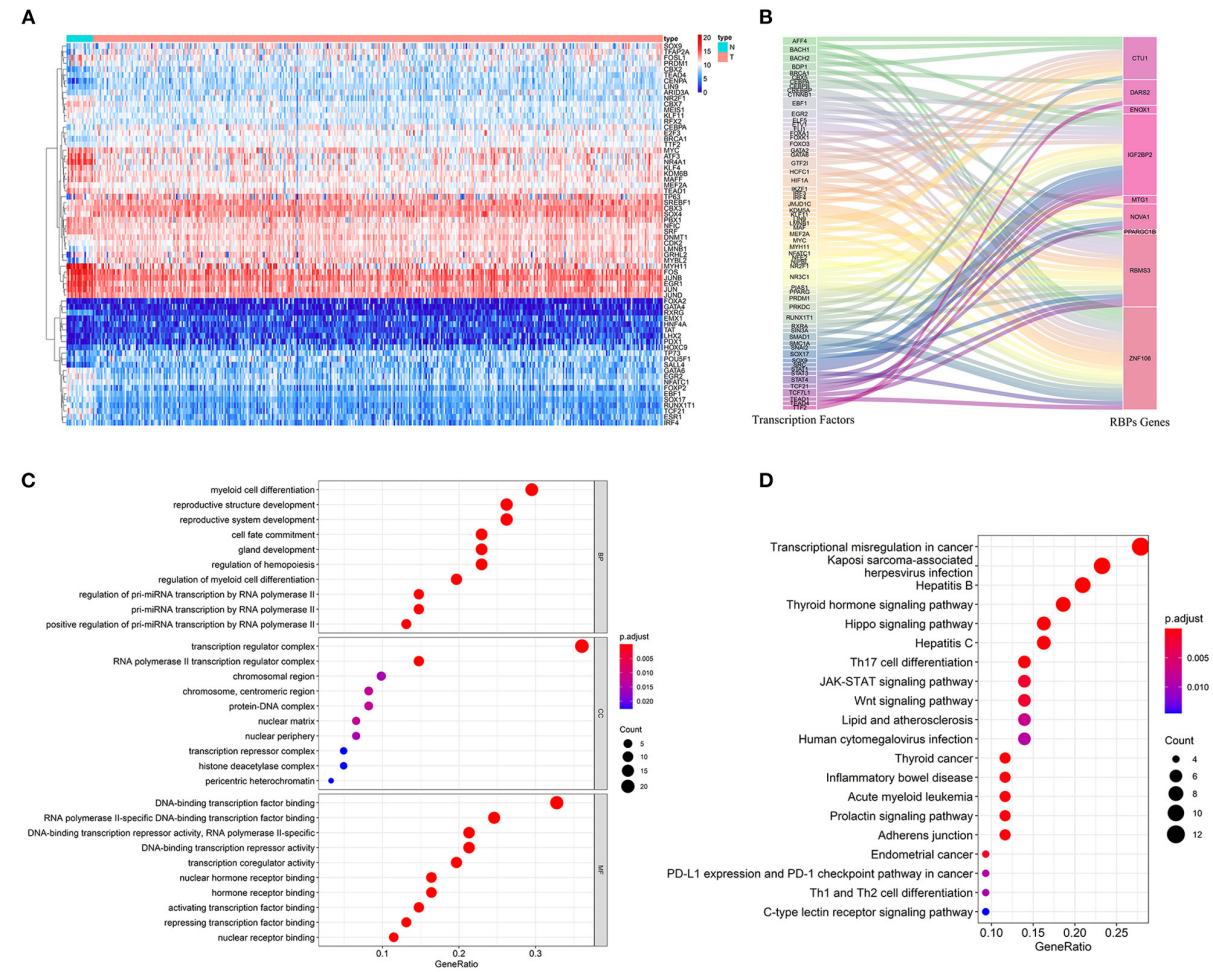
The upstream regulatory network of prognostic related RBPs. **(A)** The differentially expressed TFs in BC; **(B)** The sankey plot of TFs and RBPs regulatory networks; **(C)** GO analysis of these 61 TFs; **(D)** KEGG analysis of these 61 TFs.

To explore the role of these prognostic-related RBPs in tumorigenesis and development, we studied the upstream regulatory factors and their regulatory network. We first downloaded 318 transcription factors (TFs) associated with tumorigenesis and progression from the CISTROME project (www.cistrome.org), then screened 257 effective TFs based on the expression data of the TCGA BC patients. Figure 8A shows the differential expression distribution of these TFs in BC between tumor tissues and adjacent tissues. Next, we revealed the regulatory relationship between prognostic-related RBPs and TFs through coexpression. Sixty-one TFs were involved in regulating RBPs (Supplementary Table 2). Figure 8B shows the regulatory network of the prognostic-related RBPs and TFs. Next, we analyzed the GO and KEGG functions and pathway enrichments of these 61 TFs using the clusterProfiler package. The biological processes indicated that these TFs were significantly enriched in pri-miRNA transcription regulation by RNA polymerase II, hemopoiesis regulation, and the intracellular receptor signaling pathway. These TFs were enriched in the cellular components associated with the transcription regulator, protein-DNA, and transcription repressor complexes. In terms of molecular functions, these TFs were significantly enriched in DNA-binding transcription-factor binding, ligand-activated transcription-factor activity, and activation of transcription-factor binding (Figure 8C). KEGG analysis indicated that these TFs were significantly enriched in transcriptional misregulation in cancer, the hippo signaling pathway, Th17 cell differentiation, the JAK-STAT signaling pathway, the Wnt signaling pathway, and the PD-L1 expression and PD-1 checkpoint pathway in cancer (Figure 8D).

### Evaluation of Immune Cell Infiltration and Immunotherapy Response in BC Patients Based on the Model

**Figure 9:**
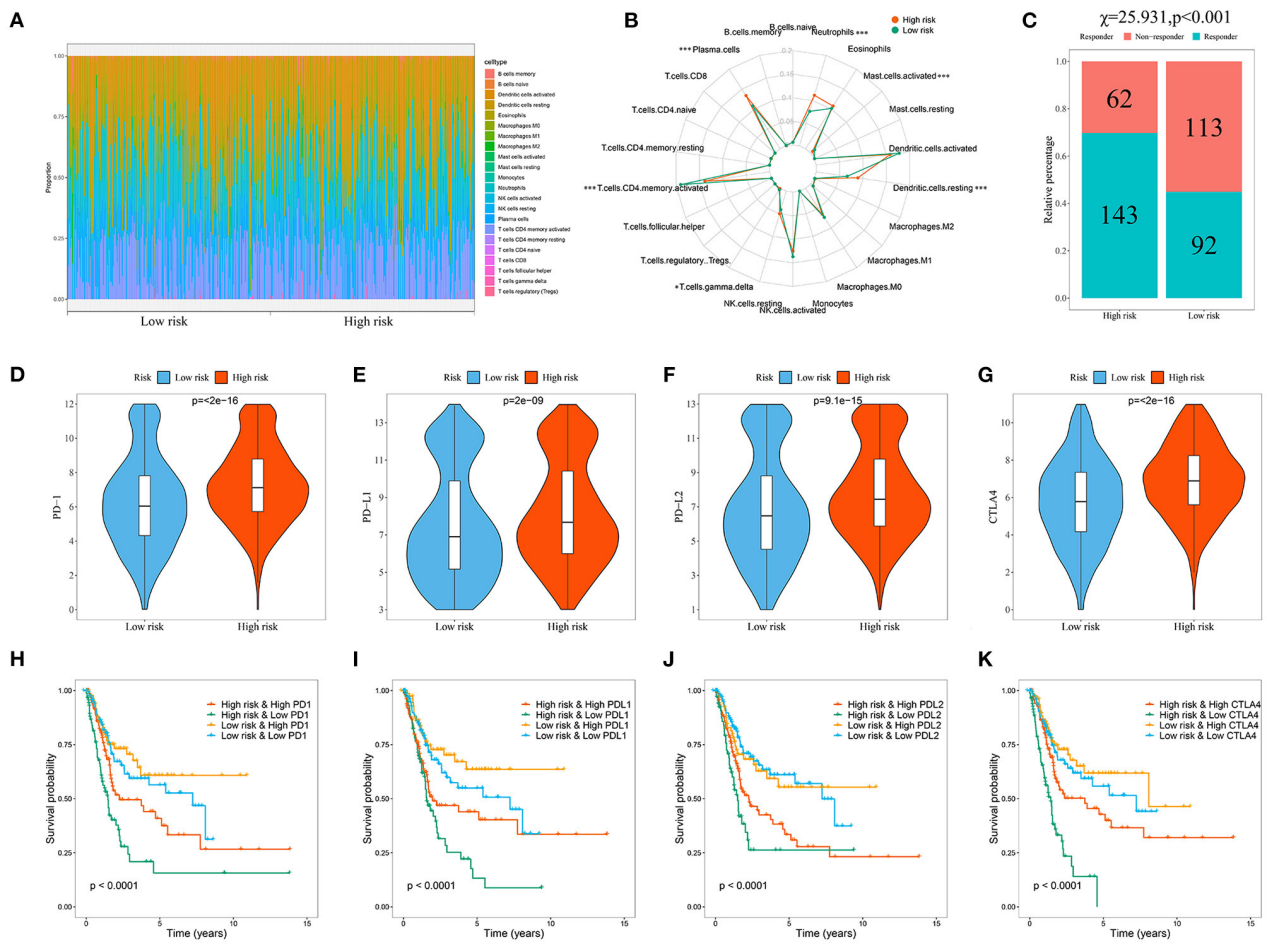
Evaluation of the immune cell infiltration and immunotherapy response in BC patients based on the model. **(A)** Landscape of immune cell infiltration in the low-risk and high-risk groups determined by the CIBERSORT algorithm; **(B)** The radar plot of 22 immune cell infiltrates in high-risk and low-risk groups; **(C)** Response rate to immunotherapy in TCGA cohort of BC patients based on TIDE algorithm; **(D–G)** Comparison of immune checkpoint gene expression levels between high- and low-risk groups; Kaplan-Meier survival curves for the four patient groups stratified by the risk score and PD-1 **(H)**, PD-L1 **(I)**, PD-L2 **(J)**, and CTLA4 **(K)**.

Because the immune microenvironment is critical to tumor occurrence, development, and treatment, we investigated the differences in immune cell infiltration between the two patient subgroups. We estimated the degree of immune cell infiltration in both subgroups by using CIBERSORT and the LM22 gene set. The CIBERSORT algorithm was conducted with 1,000 simulations. The infiltration degrees of plasma cells, CD4 memory-activated T cells, gamma-delta T cells, resting dendritic cells, activated mast cells, and neutrophils differed significantly between the two groups (Figures 9A,B), indicating that the immune cell infiltration differed between the two groups according to model risk stratification. Hence, we explored the differences in response rates to immunotherapy. Owing to the lack of a ccRCC cohort receiving immunotherapy, the tumor immune dysfunction and exclusion (TIDE) algorithm was used to preliminarily investigate the response rates of ccRCC patients in the TCGA cohort to immunotherapy. The response rate was significantly higher in the high-risk group than in the low-risk group (*P* < 0.001; Figure 9C), indicating that the model could be used as an indicator to predict immune response. Studies have shown that immune checkpoint inhibitor genes can regulate immune infiltration. Therefore, we further compared the expressions of common immune checkpoint inhibitor genes (PD-1, PD-L1, PD-L2, and CTL4) in different patient groups stratified by model to further investigate the complex interactions between immune infiltration and immune checkpoint inhibitor genes. The immune checkpoint inhibitor gene expression was significantly higher in the high-risk group than in the low-risk group (Figures 9D–G), which is consistent with previous results suggesting that the high expression of immune checkpoint inhibitor genes is associated with adverse outcomes (Sun et al., 2020). We then performed survival analysis of the patient groups stratified by model and immune checkpoint inhibitor genes to further investigate whether immune infiltration affected the clinical outcomes of patients with similar immune checkpoint gene expression levels. The OS was significantly higher in the low-risk and high immune checkpoint gene group than in the high-risk and high immune checkpoint gene group, and the patient prognoses were better in the low-risk and low immune checkpoint gene group than in the high-risk and low immune checkpoint gene group (Figures 9H–K).

### Prognostic-Related RBP Expression and Prognostic Significance Verification

**Figure 10:**
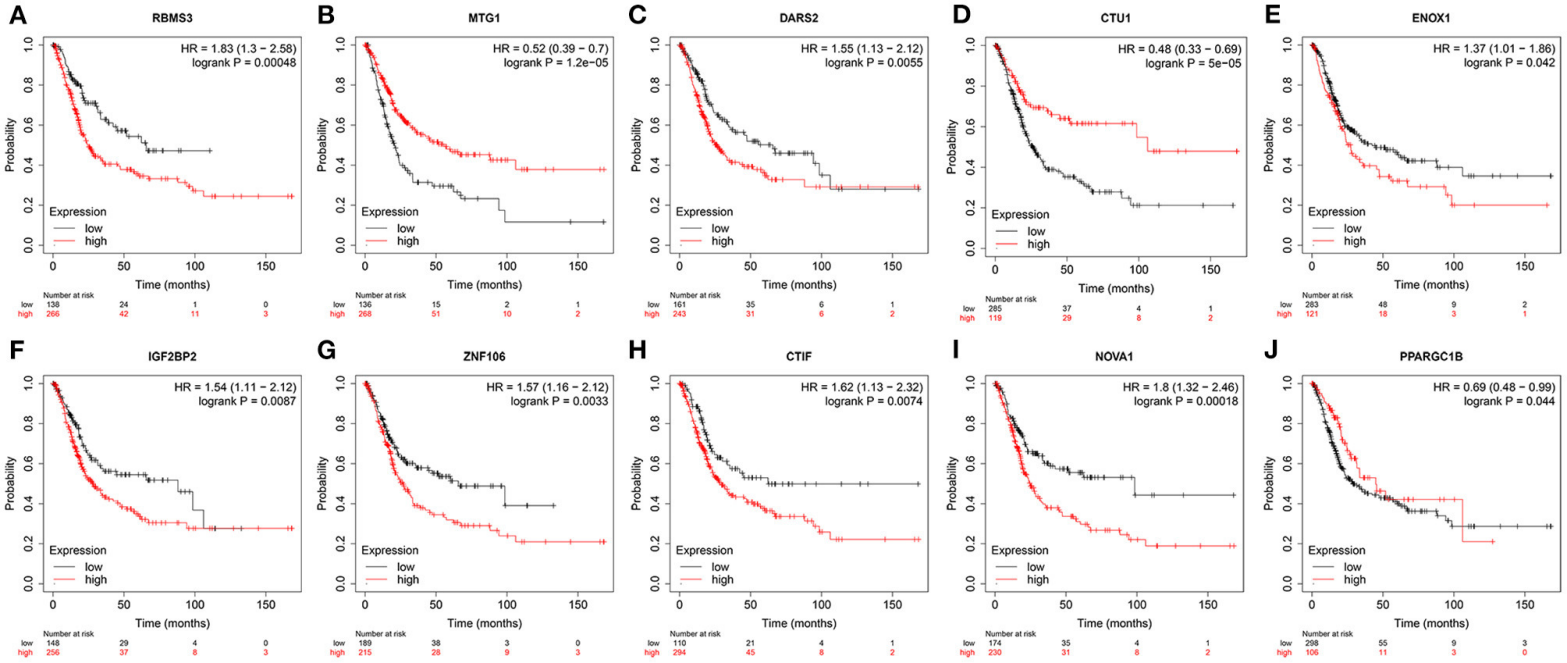
Validation the prognostic value of prognostic RBPs in BC by Kaplan-Meier plotter.

**Figure 11:**
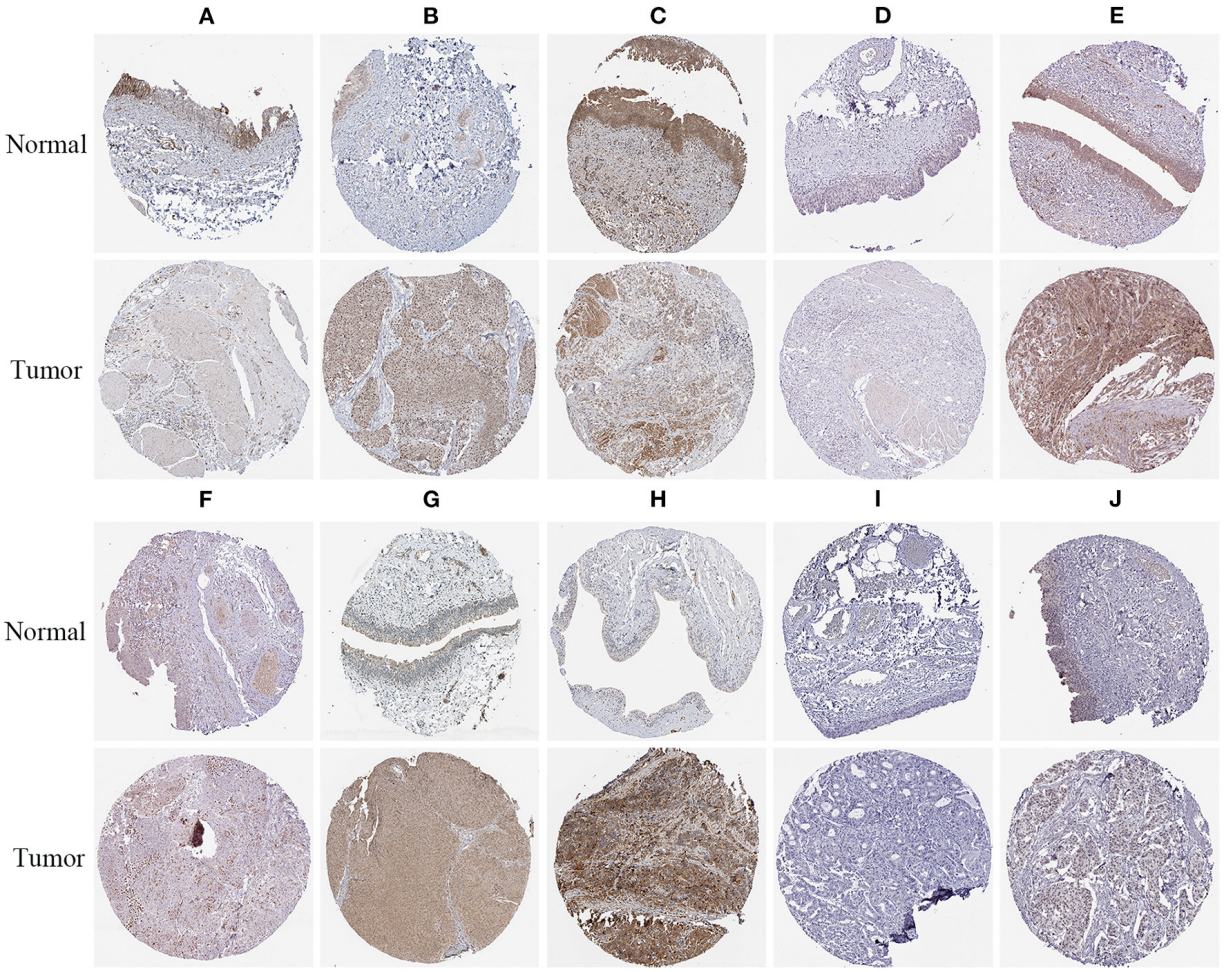
Verification of prognostic RBPs expression in BC and normal renal tissue using the HPA database. **(A)** RBMS3; **(B)** MTG1; **(C)** DARS2; **(D)** CTU1; **(E)** IGF2BP2; **(F)** ZNF106; **(G)** CTIF; **(H)** NOVA1; **(I)** TDRD1; **(J)** LIN28A.

To verify the protein expressions of the prognostic-related RBPs and their prognostic significance in patients with BC, we used the Kaplan-Meier plotter online tool to observe the correlation between the 12 RBPs and OS to further explore the prognostic values of these RBPs. The RBMS3, MTG1, DARS2, CTU1, ENOX1, IGF2BP2, ZNF106, CTIF, NOVA1, and PPARGC1B genes were related to OS in patients with BC (Figure 10). Next, we determined the immunohistochemical results of these 12 genes via the Human Protein Atlas database to determine their expression levels in BC patients. MTG1, CTU1, IGF2BP2, ZNF106, CTIF, NOVA1, and LIN28A expressions were significantly increased in BC tissues compared with those in normal bladder tissues (Figures 11B,D–H,J), whereas RBMS3 and DARS2 expressions were significantly decreased in BC tissues compared with those in normal bladder tissues (Figures 11A,C). TDRD1 expression did not significantly differ between normal bladder and BC tissues (Figure 11I).

### RT-qPCR Verification

**Figure 12:**
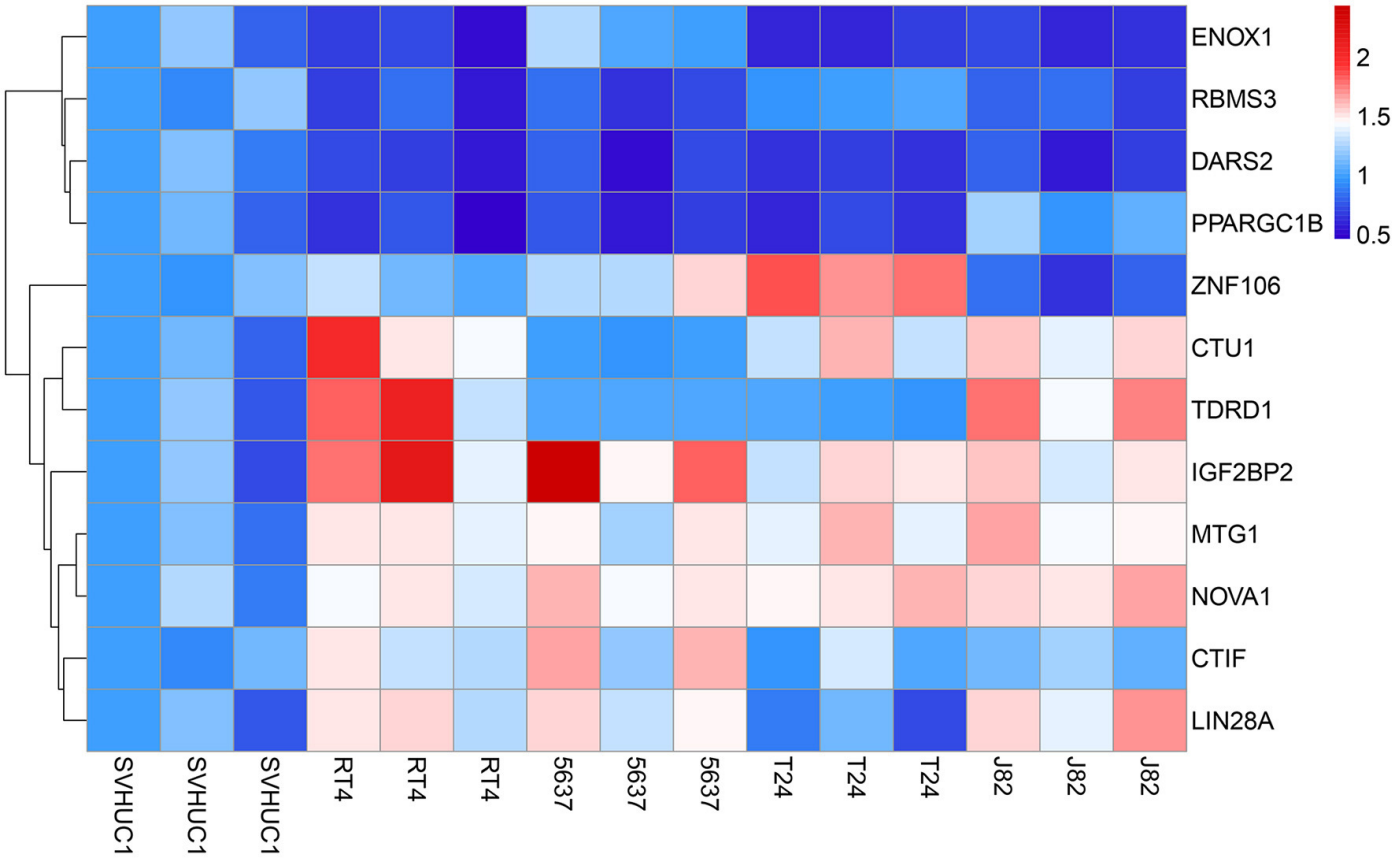
The expression heatmap of RBPs in the normal bladder epithelial cell line (SVHUC1) and bladder cancer cell lines (J82, T24, 5637, and RT4).

To further evaluate the reliability of the prognostic model, we measured the actual expression levels of these 12 RBPs in normal bladder epithelial cells and various BC cells via RT-qPCR (Figure 12). Compared with the normal bladder epithelial cell line, SVHUC1, the following results were obtained. CTIF expression was significantly upregulated in the 5637 and RT4 cell lines but did not differ in the J82 and T24 cell lines. Similarly, CTU1 expression was significantly upregulated in J82, T24, and RT4 cells but did not differ in the 5637 cells. DARS2 expression was significantly downregulated in J82, T24, 5637, and RT4 cells; ENOX1 was significantly downregulated in J82, T24, and RT4 cells, and IGF2BP2 was significantly upregulated in J82, T24, 5637, and RT4 cells. LIN28A was significantly upregulated in J82, 5637, and RT4 cells; MTG1 was significantly upregulated in J82, T24, 5637, and RT4 cells, and NOVA1 was significantly upregulated in J82, T24, 5637, and RT4 cells. PPARGC1B expression was significantly downregulated in T24, 5637, and RT4 cells, and RBMS3 was significantly downregulated in J82, 5637, and RT4 cells. TDRD1 expression was significantly upregulated in J82 and RT4 cells, and ZNF106 expression was significantly upregulated in T24 and 5637 cells but significantly downregulated in J82 cells. The RT-qPCR validation results for these cells were consistent with the bioinformatics results, thus revealing the validity and reliability of our constructed model.

### Comparison With Other RBPs-Related Prognostic Models

To determine whether our RBPs-related prognostic model was superior to other prognostic models, we compared our model with 6 RBPs (Wu et al., 2020) model and 9 RBPs model (Guo et al., 2020). Relevant prognostic genes were obtained from the corresponding literature, and survival curves and ROC curves were constructed based on the entire TCGA cohort, respectively. Through the comparative analysis of these models, we found that our model has a relatively higher prediction accuracy (Supplementary Figure 2). Moreover, we also used external cohort (GSE13507) to validate our model, which further proved its stability and applicability.

## Discussion

Post-transcriptional gene regulation is crucial for maintaining cell function, and RBPs are the most important post-transcriptional regulatory factors. RBPs participate in nearly all steps of post-transcriptional regulation, determine the fate and function of each transcript in cells, and ensure cell homeostasis (Pereira et al., 2017). RBPs have been reported to be abnormally expressed in various tumors and are associated with patient prognosis (Patry et al., 2003; King et al., 2011; Wurth et al., 2016). However, few RBPs have been studied in depth or have been found to be involved in tumorigenesis, progression, and metastasis (Abdelmohsen et al., 2008; Preca et al., 2015). Here, we systematically explored the expression patterns and roles of RBPs in patients with BC and screened 116 differentially expressed RBPs in BC and normal bladder tissues based on transcriptomic data in TCGA. We conducted GO and KEGG analyses to evaluate biological functions, then performed univariate Cox regression, LASSO regression, and multivariate Cox regression analyses to screen prognostic-related RBPs and assess their prognostic significance. We also established a risk score model to predict the prognoses of patients with BC based on these prognostic genes. Moreover, we combined risk score with other clinical variables to conduct a nomogram to establish a quantitative prognostic evaluation method for patients with BC.

Enrichment analysis of biological functions and pathways of the differentially expressed RBPs indicated that these RBPs were significantly enriched in mRNA processing, posttranscriptional regulation of gene expression, regulation of cellular amide metabolic processes, regulation of mRNA metabolic processes, RNA catabolic processes, gene silencing, RNA splicing, regulation of gene expression, epigenetics, DNA modification, nucleic acid phosphodiester bond hydrolysis, ribonucleoprotein granules, nucleolar parts, the telomerase holoenzyme complex, mRNA binding, catalytic activity, acting on RNA, translation regulator activity, AU-rich-element binding, translation factor activity, RNA binding, single-stranded RNA binding, double-stranded RNA binding, pre-mRNA binding, nuclease activity, and ribonucleoprotein-complex binding.

Recent studies have shown that regulating translation and RNA processing and metabolism affect the occurrence and progression of many diseases (Jain et al., 2019; Kim et al., 2019; Siang et al., 2020). Here, we conducted a systematic analysis and identified a signature of 12 prognosis-associated RBPs: RBMS3, MTG1, DARS2, CTU1, ENOX1, IGF2BP2, ZNF106, CTIF, NOVA1, PPARGC1B, TDRD1, and LIN28A. RBMS3 is a member of the single-stranded binding protein family of the c-myc gene and mainly encodes a glycine-rich RBP. We found that mRNA levels of RBMS3 were significantly reduced in BC tissues; this was similar to the results of Zhu et al. (2019), who found that RBMS3 expression was downregulated and associated with a poor prognosis in patients with breast cancer. Additionally, downregulated RBMS3 expression is associated with poor prognosis in lung squamous cell carcinoma, gastric cancer and esophageal squamous cell carcinoma (Li et al., 2011a; Liang et al., 2015; Zhang T. et al., 2016), and downregulation of RBMS3 in ovarian cancer increased chemotherapeutic resistance (Wu et al., 2019). MTG1 is a conserved ribosomal assembly guanosine triphosphatase, which functions as a cofactor of mitoribosome. As shown in our study, MTG1 mRNA was significantly increased in BC tissues. Faraj Shaglouf et al. (2020) found that MTG1 expression was increased in hepatocellular carcinoma and played a regulatory role in its progression. Liu and Pan (2016) found that MTG1 played an important role in tumor induction or progression. DARS2 mainly encodes mitochondrial tRNA synthase, which is crucial for mitochondrial folding protein reactions. DARS2 gene mutation was reported to be related to leukoencephalopathy (Köhler et al., 2015). Qin et al. (2017) found that DARS2 expression was upregulated in hepatocellular carcinoma and that DARS2 regulated cell cycle progression and apoptosis of hepatocellular carcinoma cells. CTU1 is mainly involved in modifying the swinging position of U34 in some tRNAs. CTU1 expression was also upregulated in melanoma cells (Rapino et al., 2018). Delaunay et al. (2016) found that CTU1 expression was upregulated in breast cancer cells, and deleting the CTU1 gene significantly reduced migration and tumorsphere formation in breast cancer cells. ENOX1 is a copper-binding protein expressed in endothelial and other cells and has NADH oxidase activity and promotes angiogenesis. Studies have shown that inhibiting ENOX1 activity reduced the ability of endothelial cells to migrate and form tubular structures (Geng et al., 2019). Therefore, targeted inhibition of ENOX1 activity to inhibit tumor angiogenesis may be a feasible strategy for tumor control. Smith et al. (2016) found that targeted inhibition of ENOX1 in tumor stroma improved radiotherapeutic efficacy in tumor patients. IGF2BP2 is a member of the insulin-like growth factor 2 mRNA-binding protein family, which are newly reported m6A “readers.” IGF2BP2 is primarily responsible for the stability of targeted mRNA and is associated with thousands of targets, including MYC, KRAS, and MDR1 (Xiao et al., 2016). Previous studies revealed that IGF2BP2 was upregulated in multiple tumors and was associated with tumor growth, migration, and energy metabolism (Li et al., 2015). IGF2BP2 overexpression was associated with decreased cell adhesion and migration in breast cancer cells (Li et al., 2015). Another study found that IGF2BP2 expression was associated with tumor progression in glioblastomas and hepatomas (Cao et al., 2018). ZNF106 has a variety of cellular functions, including insulin receptor signaling, rRNA transcriptional regulation, and maintenance of testicular development, and is essential for maintaining motor and sensory neurons (Joyce et al., 2016). In this study, ZNF106 mRNA levels were significantly downregulated in BC tissues. However, the role of ZNF106 in tumors is unreported. CTIF is a specific factor involved in the first round of translation driven by a nuclear cap-binding protein complex, which contributes to protein and mRNA quality control. We found that CTIF mRNA levels were significantly downregulated in BC tissues. CTIF also shares a domain with inclusion bodies containing the SOD1 mutant G93A, which is a histologic feature of amyotrophic lateral sclerosis; thus, CTIF may be related to occurrence and development of this disease (Park et al., 2018). NOVA1 is a pre-mRNA-binding splicing factor expressed in the central nervous system and is necessary for motor system development. Studies have reported that NOVA1 plays a key role in various tumors such as gastric cancer, astrocytoma, liver cancer and lymphoma (Kim et al., 2016, 2017). Zhang Y. et al. (2016) found that NOVA1 overexpression promoted the growth of hepatocellular carcinoma. Shen et al. (2015) found that miR-339 inhibited gastric cancer cell growth, invasion, migration, and tumorigenicity by regulating NOVA1 expression. PPARGC1B is a co-activator of the oxisome proliferator-activated receptor and an important regulator of energy metabolism. Li et al. (2011b) found a positive correlation between PPARGC1B polymorphism and the risk of ER-positive breast cancer. Eichner et al. (2010) found that miR378 was embedded in PPARGC1B, and its expression was associated with human breast cancer progression. TDRD1 belongs to a large family of proteins containing the Tudor domain, which is specific to germ cells. In our study, TDRD1 mRNA expression was elevated in BC tissues; this was similar to the results of Boormans et al. (2013), who found that TDRD1 was upregulated in prostate cancer with ERG overexpression. Brase et al. (2011) found that TDRD1 expression was upregulated in both ERG-negative and ERG-positive prostate cancer. LIN28A is a pluripotent factor and highly conserved RNA-binding protein associated with neurodevelopment and the pathogenesis of various advanced cancers (Viswanathan et al., 2009). In our study, LIN28A mRNA expression was increased in BC tissues, which was similar to the findings of Huang et al., who found that LIN28A expression was increased in thyroid papillary carcinoma tissues and cells. This was associated with higher tumor stages and lymph node metastasis, whereas LIN28A knockdown suppressed tumor cell proliferation, invasion, and migration (Huang et al., 2018). A recent study suggested that LIN28A played a role in the mechanism of resistance to paclitaxel in patients with breast cancer (Lv et al., 2012).

We used multiple stepwise Cox regression analysis to establish a risk score model for predicting BC patient prognoses based on these 12 genes. Survival and ROC curve analyses showed that these 12 genes had good diagnostic ability and could be used to screen out BC patients who had poor prognoses. However, the specific molecular mechanisms of these 12 RBPs in BC remain unclear, and the underlying molecular mechanisms should be explored. Subsequently, we assessed the relationship between risk score model and clinical variables and between prognostic-related RBPs and clinical variables and found that the risk score model was significantly associated with clinical progression. We then established a nomogram to more intuitively predict 1-year, 3-year, and 5-year survival estimates in patients with BC. TCGA and GEO data were used to evaluate and verify the nomogram performance. We also analyzed the regulatory relationship between these prognostic-related RBPs and their upstream regulators and identified 61 TFs that may help regulate BC. TFs are the largest family of proteins involved in transferring genetic information from DNA to mRNA (Levine and Tjian, 2003). TFs are involved in regulating complex molecular mechanisms in many diseases, including tumors, and play important roles in tumor growth, invasion and metastasis. Many studies have found that BC plays a key role in tumor growth, invasion and metastasis (Lambert et al., 2018). Schulte et al. (2012) found a positive correlation between cytoplasmic and nuclear expressions of TFs and activated mesenchymal fibroblasts, which may be involved in the invasive phenotype of BC. Huaqi et al. (2019) showed that SOX18 played a procancer role in BC and may be a potential prognostic biomarker and therapeutic target for BC. Therefore, the specific roles and mechanisms of these TFs in BC deserve further study.

Next, we further analyzed the differences in immune cell infiltration and response rates to immunotherapy among different groups of patients with BC based on the model. Our results revealed differences in immune cell infiltration, immune response, and immune checkpoint inhibitor gene expression levels between the groups. BC is an immunosensitive tumor infiltrated by various immune cells (Schwamborn et al., 2019). Many studies have reported the influence of the immune microenvironment on BC occurrence and immunotherapy, including the long-term application of *Bacillus subtilis* Calmette-Guerin and PD-1/PD-L1 blockers in treating BC (Eich et al., 2019). Tumor-infiltrating immune cells are the main components of the tumor microenvironment and are closely related to the efficacy and clinical results of targeted drugs (Liu et al., 2018). Thus, our model can be used as an indicator to predict immune cell infiltration and immune response in patients with BC.

We used the Kaplan-Meier plotter online tool to study the prognostic significance of the 12 RBP-encoding genes and found that MTG1, CTU1, and PPARGC1B gene expressions were related to good prognoses in patients with BC, while high RBMS3, DARS2, ENOX1, IGF2BP2, ZNF106, CTIF, and NOVA1 gene expressions were related to poor prognoses. We further confirmed the expressions of these genes at the cellular level. The results suggested that the signatures of these 12 genes may help modulate treatment, assess treatment outcomes and predict patient survival.

Overall, this study was conducted to understand the BC pathogenesis, progression, invasion and metastasis from a new perspective. Our predictive model well-predicted the survival times of patients with BC, suggesting that the signatures of these 12 genes have an important biological background, which may be helpful for clinical adjuvant therapy.

Our study had some limitations. First, we used bioinformatics techniques to evaluate the diagnostic and prognostic prediction values of several key RBPs in BC. However, the specific functions and mechanisms of these key RBPs in BC growth and progression remain unclear, and further *in vitro* and *in vivo* experiments are needed. Second, our risk score model should be verified with a multicenter, large prospective cohort of patients with BC. Finally, our study was based only on bio-omics data, and analyzing different patient characteristics on different platforms can lead to patient heterogeneity.

In conclusion, we systematically analyzed the biological functions and prognostic values of differentially expressed RBPs in BC using bioinformatics techniques. These RBPs may play important roles in BC tumor occurrence, progression, invasion and metastasis. Additionally, we first constructed a prognostic model of BC based on features of 12 RBPs, suggesting that the model can be used as an independent prognostic factor of BC. These findings provide new insights into the mechanisms by which BC occurs and progresses and may aid in developing new clinical therapeutic targets or prognostic markers.

## Data Availability Statement

Transcriptome data of 19 normal bladder tissue samples and 411 bladder cancer tissue samples were downloaded from The Cancer Genome Atlas database (TCGA, https://portal.gdc.cancer.gov/).

## Author Contributions

YW designed the study and performed the data analysis. ZheL, XW, HF, BH, and BL performed the data analysis. YL, YR, XL, ZhuL, SW, and JL performed the data analysis and revised the manuscript. TW designed the study and revised the manuscript. All authors read and approved the final manuscript.

## Conflict of Interest

The authors declare that the research was conducted in the absence of any commercial or financial relationships that could be construed as a potential conflict of interest.

## Abbreviations

BC: Bladder cancer
TCGA: The Cancer Genome Atlas
KEGG: Kyoto Encyclopedia of Genes and Genomes
GO: Gene ontology
FC: Fold change
OS: Overall survival
DFS: Disease free survival
LASSO: Least absolute shrinkage and selection operator
ROC: Receiver operating characteristic
AUC: Area under the receiver operating characteristic curve
FDR: False discovery rate
TFs: Transcription factors
TIDE: The Tumor Immune Dysfunction and Exclusion.
