# Supplementary material for: Identification of the Functions and Prognostic Values of RNA Binding Proteins in Bladder Cancer
Source: Front Genet. 2021 Jun 22;12:574196. doi: 10.3389/fgene.2021.574196 (PMC8258248; doi:10.3389/fgene.2021.574196)
Supplement: Supplementary file 1 [file Table_1.DOCX]

| Name | Primer | Sequence | Size |
| --- | --- | --- | --- |
| Human GAPDH | Forward | 5‘- GAGAAGGCTGGGGCTCATTT-3’ | 231bp |
|  | Reverse | 5‘- AGTGATGGCATGGACTGTGG-3’ |  |
| Human CTIF | Forward | 5‘- AAGGTACCGGATGAAGTGGC-3’ | 156bp |
|  | Reverse | 5‘- GGAGTTGGTGGAGTTCTGGG-3’ |  |
| Human CTU1 | Forward | 5‘- GGAGCGTGACTTGATGGACA-3’ | 102bp |
|  | Reverse | 5‘- TGGAGACGAAATGAGGGTGC-3’ |  |
| Human DARS2 | Forward | 5‘- GTGGTCCTACTAACTGCTGGA-3’ | 119bp |
|  | Reverse | 5‘- AACAGAGTGGGGTCACGGAG-3’ |  |
| Human ENOX1 | Forward | 5‘- TACTCACACAGGCTTCCAGC-3’ | 141bp |
|  | Reverse | 5‘- CTCCTCCTTTGCTTCTCCCG-3’ |  |
| Human IGF2BP2 | Forward | 5‘- CGGGGAAGAGACGGATGATG-3’ | 150bp |
|  | Reverse | 5‘- GGTAGTCCACGAAGGCGTAG-3’ |  |
| Human LIN28A | Forward | 5‘- AGTAAAGAGATGCAATTGGGGG -3’ | 106bp |
|  | Reverse | 5‘- ACCTGCATAAAAGTTCCTGAGTG -3’ |  |
| Human MTG1 | Forward | 5‘- AGAACTTCCCCCTGTGCGG-3’ | 177bp |
|  | Reverse | 5‘- GCTTAAGCCCAAGGGTTTCC-3’ |  |
| Human NOVA1 | Forward | 5‘- CCACACTGGGGTTCCCATAG-3’ | 117bp |
|  | Reverse | 5‘- AGAAAATACTGGCCGTCGCC-3’ |  |
| Human PPARGC1B | Forward | 5‘- CCCTGAAGATGACGTGGGTC -3’ | 169bp |
|  | Reverse | 5‘- GAGCTTCTGCAGCAGTGAGA -3’ |  |
| Human RBMS3 | Forward | 5‘- GCAAGGATTCGGAGTTGTGC-3’ | 166bp |
|  | Reverse | 5‘- CACACACAGCCTTCCCAGAT-3’ |  |
| Human TDRD1 | Forward | 5‘- GGATTGGAGGCGAGGGAAG-3’ | 132bp |
|  | Reverse | 5‘- CATCGAGGCCTGGAAAGAGG-3’ |  |
| Human ZNF106 | Forward | 5‘- GGCTGTGAATGAGATGGACG-3’ | 149bp |
|  | Reverse | 5‘- TGGACAGATCAGGTTCCCCA-3’ |  |

Supplementary Table S1. The sequences of primer RNA.
