# Supplementary material for: Identification of the Functions and Prognostic Values of RNA Binding Proteins in Bladder Cancer
Source: Front Genet. 2021 Jun 22;12:574196. doi: 10.3389/fgene.2021.574196 (PMC8258248; doi:10.3389/fgene.2021.574196)
Supplement: Supplementary file 2 [file Table_2.docx]

Table S2. The regulatory relationship between transcription factors and RBPs.

| Transcription factors | RNA binding proteins genes | Coefficient | p-value | Regulation |
| --- | --- | --- | --- | --- |
| AFF4 | CTU1 | -0.46216 | 4.36E-23 | Negative |
| AFF4 | ZNF106 | 0.498736 | 3.60E-27 | Positive |
| BACH1 | CTU1 | -0.41915 | 7.11E-19 | Negative |
| BACH1 | ZNF106 | 0.542383 | 9.84E-33 | Positive |
| BACH2 | IGF2BP2 | 0.487547 | 7.22E-26 | Positive |
| BACH2 | RBMS3 | 0.525508 | 1.74E-30 | Positive |
| BDP1 | CTU1 | -0.4256 | 1.81E-19 | Negative |
| BDP1 | ZNF106 | 0.505079 | 6.27E-28 | Positive |
| BRCA1 | DARS2 | 0.483489 | 2.08E-25 | Positive |
| CBX5 | ZNF106 | 0.416325 | 1.28E-18 | Positive |
| CEBPA | IGF2BP2 | -0.47581 | 1.49E-24 | Negative |
| CEBPB | IGF2BP2 | 0.426649 | 1.45E-19 | Positive |
| CREBBP | ZNF106 | 0.420452 | 5.41E-19 | Positive |
| CTNNB1 | ZNF106 | 0.403566 | 1.72E-17 | Positive |
| EBF1 | ENOX1 | 0.420988 | 4.83E-19 | Positive |
| EBF1 | NOVA1 | 0.525915 | 1.54E-30 | Positive |
| EBF1 | RBMS3 | 0.647084 | 5.23E-50 | Positive |
| EGR2 | IGF2BP2 | 0.424934 | 2.09E-19 | Positive |
| EGR2 | RBMS3 | 0.50021 | 2.41E-27 | Positive |
| ELF5 | IGF2BP2 | -0.44514 | 2.39E-21 | Negative |
| ETV1 | RBMS3 | 0.489582 | 4.22E-26 | Positive |
| FLI1 | RBMS3 | 0.580446 | 2.69E-38 | Positive |
| FOXA1 | IGF2BP2 | -0.54788 | 1.71E-33 | Negative |
| FOXK1 | ZNF106 | 0.523355 | 3.30E-30 | Positive |
| FOXO3 | CTU1 | -0.41813 | 8.81E-19 | Negative |
| FOXO3 | ZNF106 | 0.45912 | 9.07E-23 | Positive |
| GATA2 | IGF2BP2 | -0.4183 | 8.50E-19 | Negative |
| GATA6 | RBMS3 | 0.499533 | 2.90E-27 | Positive |
| GTF2I | CTU1 | -0.45209 | 4.79E-22 | Negative |
| GTF2I | DARS2 | 0.422182 | 3.75E-19 | Positive |
| GTF2I | ZNF106 | 0.487161 | 7.99E-26 | Positive |
| HCFC1 | ZNF106 | 0.422956 | 3.18E-19 | Positive |
| HIF1A | CTU1 | -0.41269 | 2.72E-18 | Negative |
| HIF1A | MTG1 | -0.40145 | 2.62E-17 | Negative |
| HIF1A | ZNF106 | 0.441046 | 6.06E-21 | Positive |
| IKZF1 | RBMS3 | 0.455756 | 2.02E-22 | Positive |
| IRF3 | CTU1 | 0.401113 | 2.80E-17 | Positive |
| IRF4 | RBMS3 | 0.40047 | 3.17E-17 | Positive |
| JMJD1C | CTU1 | -0.48674 | 8.93E-26 | Negative |
| JMJD1C | ZNF106 | 0.533279 | 1.66E-31 | Positive |
| KDM5A | ZNF106 | 0.494713 | 1.07E-26 | Positive |
| KLF11 | ZNF106 | 0.430096 | 6.86E-20 | Positive |
| LIN9 | DARS2 | 0.454212 | 2.91E-22 | Positive |
| LMNB1 | DARS2 | 0.416005 | 1.37E-18 | Positive |
| MAF | RBMS3 | 0.534469 | 1.16E-31 | Positive |
| MEF2A | CTU1 | -0.44174 | 5.18E-21 | Negative |
| MEF2A | ZNF106 | 0.536231 | 6.71E-32 | Positive |
| MYC | IGF2BP2 | 0.455019 | 2.41E-22 | Positive |
| MYH11 | RBMS3 | 0.536101 | 6.99E-32 | Positive |
| MYH11 | NOVA1 | 0.550116 | 8.32E-34 | Positive |
| NFATC1 | RBMS3 | 0.483266 | 2.21E-25 | Positive |
| NFE2 | IGF2BP2 | 0.46167 | 4.91E-23 | Positive |
| NIPBL | ZNF106 | 0.430319 | 6.53E-20 | Positive |
| NR2F1 | NOVA1 | 0.471372 | 4.54E-24 | Positive |
| NR3C1 | MTG1 | -0.44109 | 6.00E-21 | Negative |
| NR3C1 | RBMS3 | 0.456085 | 1.87E-22 | Positive |
| NR3C1 | ZNF106 | 0.468637 | 8.96E-24 | Positive |
| NR3C1 | IGF2BP2 | 0.489166 | 4.71E-26 | Positive |
| PIAS1 | ZNF106 | 0.48578 | 1.15E-25 | Positive |
| PPARG | IGF2BP2 | -0.53081 | 3.53E-31 | Negative |
| PRDM1 | RBMS3 | 0.474215 | 2.23E-24 | Positive |
| PRDM1 | IGF2BP2 | 0.481568 | 3.42E-25 | Positive |
| PRKDC | ZNF106 | 0.491318 | 2.66E-26 | Positive |
| PRKDC | DARS2 | 0.501884 | 1.52E-27 | Positive |
| RUNX1T1 | ENOX1 | 0.525242 | 1.88E-30 | Positive |
| RUNX1T1 | NOVA1 | 0.53222 | 2.30E-31 | Positive |
| RUNX1T1 | RBMS3 | 0.577802 | 6.93E-38 | Positive |
| RXRA | PPARGC1B | 0.431982 | 4.54E-20 | Positive |
| SIN3A | ZNF106 | 0.437493 | 1.34E-20 | Positive |
| SMAD1 | CTU1 | -0.44127 | 5.76E-21 | Negative |
| SMAD1 | ZNF106 | 0.529392 | 5.42E-31 | Positive |
| SMC1A | ZNF106 | 0.424641 | 2.22E-19 | Positive |
| SNAI2 | IGF2BP2 | 0.440349 | 7.09E-21 | Positive |
| SOX17 | RBMS3 | 0.404593 | 1.40E-17 | Positive |
| SOX17 | NOVA1 | 0.45899 | 9.36E-23 | Positive |
| SOX9 | IGF2BP2 | 0.454239 | 2.89E-22 | Positive |
| SRC | IGF2BP2 | -0.45845 | 1.07E-22 | Negative |
| STAT1 | IGF2BP2 | 0.470948 | 5.05E-24 | Positive |
| STAT3 | ZNF106 | 0.473999 | 2.35E-24 | Positive |
| STAT4 | RBMS3 | 0.422786 | 3.30E-19 | Positive |
| STAT4 | IGF2BP2 | 0.496545 | 6.54E-27 | Positive |
| TCF21 | NOVA1 | 0.519478 | 1.03E-29 | Positive |
| TCF7L1 | IGF2BP2 | 0.434758 | 2.47E-20 | Positive |
| TCF7L1 | RBMS3 | 0.439877 | 7.89E-21 | Positive |
| TEAD1 | ZNF106 | 0.492735 | 1.82E-26 | Positive |
| TEAD4 | IGF2BP2 | 0.652416 | 4.50E-51 | Positive |
| TTF2 | DARS2 | 0.444093 | 3.04E-21 | Positive |
