# Supplementary figures and images for: Identification of the Functions and Prognostic Values of RNA Binding Proteins in Bladder Cancer
Source: Front Genet. 2021 Jun 22;12:574196. doi: 10.3389/fgene.2021.574196 (PMC8258248; doi:10.3389/fgene.2021.574196)

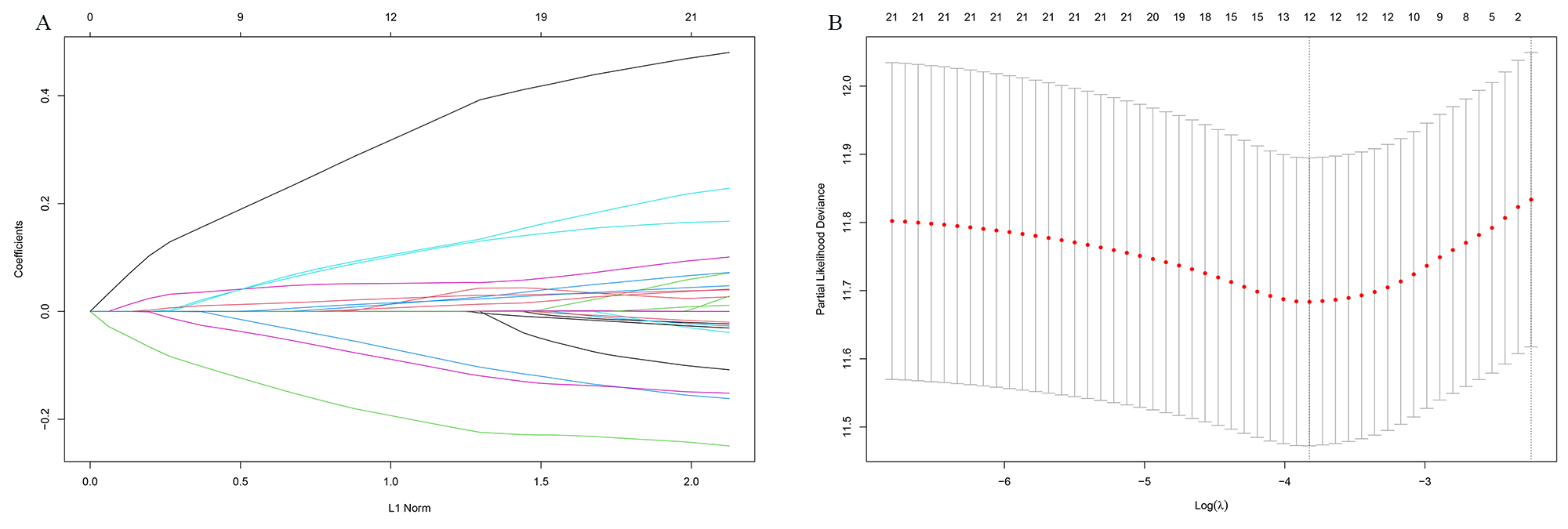

Supplement: Supplementary file 3 [file Image_1.TIF]

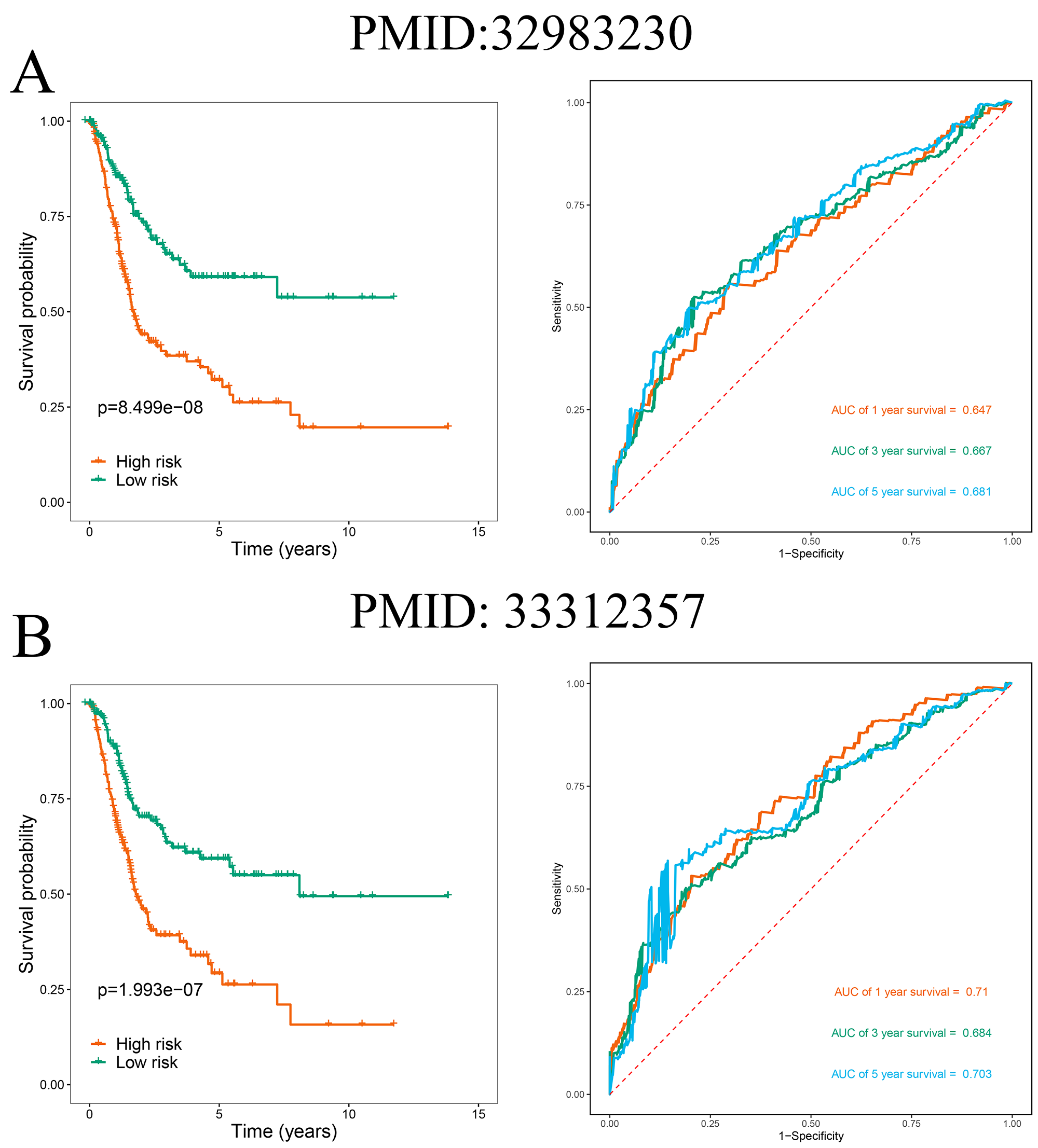

Supplement: Supplementary file 4 [file Image_2.TIF]
